# Global, Regional, and National Burden of Low Bone Mineral Density From 1990 to 2019: Results From the Global Burden of Disease Study 2019

**DOI:** 10.3389/fendo.2022.870905

**Published:** 2022-05-24

**Authors:** Yimin Dong, Honglei Kang, Renpeng Peng, Kehan Song, Qian Guo, Hanfeng Guan, Meipeng Zhu, Dawei Ye, Feng Li

**Affiliations:** ^1^ Department of Orthopedics, Tongji Hospital, Tongji Medical College, Huazhong University of Science and Technology, Wuhan, China; ^2^ Cancer Center, Tongji Hospital, Tongji Medical College, Huazhong University of Science and Technology, Wuhan, China

**Keywords:** low bone mineral density, osteoporosis, GBD 2019, mortality, disability-adjusted life year, burden

## Abstract

**Objective:**

We aim to explore the global spatial prevalence and temporal trends of the burden of low bone mineral density (LBMD) worldwide, due to a lack of related studies.

**Design:**

Cross-sectional study.

**Methods:**

We used data from the Global Burden of Disease Study 2019 to conduct this study. LBMD in the GBD study includes both osteopenia and osteoporosis. The estimation for the prevalence, measured by the summary exposure value (SEV), and burden of LBMD was made in DisMod-MR 2.1, a Bayesian meta-regression tool. Correlation analysis was performed using the Spearman rank order correlation methods. The temporal trends were represented by the estimated annual percentage change (EAPC).

**Results:**

In 2019, there were 438 thousand deaths and 16.6 million DALYs attributable to LBMD, increasing by 111.1% and 93.8% respectively, compared to that in 1990. From 1990 to 2019, the prevalence of LBMD has decreased worldwide, but has increased in high-income North America. Some countries, such as the United States, Australia, Canada, and China had increased disability and mortality rates of LBMD with time. Countries with low socio-demographic index (SDI) had higher incidence and mortality rate than those with high SDI. The prevalence of LBMD was lower in males, but the attributable disability and mortality were higher in males in all years from 1990 to 2019.

**Conclusion:**

With population aging, countries worldwide, especially those with low-SDI, will face increasing challenges in reducing the burden attributable to LBMD and osteoporosis. The treatment of osteoporosis has been overlooked in men for a long time. Effective measures are warranted to control the prevalence and burden of LBMD.

## Introduction

Low bone mineral density (LBMD) is a state of decreased bone mass caused by either increased bone resorption or attenuated bone formation, or both. When this condition progresses, substantial changes will occur in the microarchitecture and mechanical properties of bone, reducing bone strength that predisposes patients to the risk of various fractures, such as hip or vertebral fractures. Decreased bone density has two stages, Osteopenia and osteoporosis, which are usually diagnosed according to the SD scores of peak bone mineral density value from healthy young women (1). Osteopenia has a T-score between -1 and -2.5, while osteoporosis is defined as a T-score equal to or less than -2.5. BMD is mainly measured in the spine or the femoral neck by dual-energy X-ray absorptiometry (2), and the BMD measured at the femoral neck has higher predicting value in terms of assessing the risk of hip fracture (3).

The epidemiology of LBMD and the resultant fractures have been reported in different countries and time. It is estimated that approximately half of women suffer at least one bone fracture after menopause (4). Osteoporosis causes about 8.9 million fractures globally and 20–25% occur in men every year (5). In France, over 170 thousand patients were admitted to hospital due to osteoporotic fractures in 2013 (6). It also affected about 10% of people aged over 40 years in 2005 in Japan, and the absolute number affected was estimated to be 12.8 million, with about 75% female cases (7). In the same year, osteoporosis-related fractures were predicted to be over 2 million in the USA (8).

Most studies about the burden and cost attributable to LBMD or osteoporosis are based on restricted data, or are limited to regional levels. The Global Burden of Diseases (GBD) study provides a chance to analyze the incidence, prevalence and burden attributable to diseases, injuries or risks in the global level (9, 10). Diseases in the GBD study include communicable, noncommunicable and malignant diseases (11–15), while LBMD is defined as a risk factor that predisposes patients to higher risk of various fracture outcomes, including fractures of the hip, vertebrae and many other sites. LBMD in this study includes both osteopenia and osteoporosis. We reported the prevalence and burden of LBMD worldwide, using data of the GBD study 2019. We also demonstrated the trends of prevalence and burden from 1990 to 2019, and analyzed their association with the socio-demographic index (SDI) to provide a general understanding about the current burden of LBMD.

## Materials and Methods

### Data Source and Definition

The GBD study 2019 was used to obtain all the data for this study. Detailed data source and methodology of data processing have been clearly introduced elsewhere (16, 17). The final data for each disease, injury, or risk factor can be interpreted in the context of location, year and age groups. We focused on the global prevalence and attributable burden of LBMD. According to the parent GBD risk factor study, BMD was measured at the femoral neck by dual-energy X-ray absorptiometry. LBMD was determined according to the difference between the BMD of a specific population and the 99th percentile of a reference population of the same age and sex (18). In this study, LBMD includes both osteopenia and osteoporosis, two conditions of decreased bone strength, compared to normal bone mineral density. The input data for estimating the prevalence of LBMD were based on a systematic review in GBD 2010 on population-based studies, which have been updated for GBD 2013 and 2015. To estimate the attributable burden of LBMD, the GBD 2019 risk factor study followed a series of steps: determination of the risk-outcome pairs; relative risk estimation; estimation of exposure levels and distributions; Determination of the theoretical minimum-risk exposure level; and computation of the attributable burden. Detailed methodology for the estimations of all risk factors can be learned from the parent GBD 2019 risk factor study (17). Here we summarized the methods for these steps specific to LBMD.

### Risk-Outcome Pairs for LBMD

The GBD 2019 has included 87 risk factors associated with the incidence and burden of diseases or injuries. LBMD is a kind of such risk that predisposes patients at a high risk of fracture outcomes, including fractures of the hip, vertebrae, clavicle, scapula, humerus, skull, sternum, face bone, radius or ulna, femur, patella, tibia, fibula, ankle, and pelvis. In GBD 2019, LBMD along with these fractures are defined as risk-outcome pairs. These outcomes can also be understanded as nature-of-injuries in the study, which are caused by cause-of-injuries, such as falls, road injuries, conflict and terrorism. LBMD-attributable burden was estimated as the disability or mortality caused by fractures (17). The primary data to estimate fracture-related disability were obtained from hospital medical records, insurance claims, emergency department records, while the methods have also been summarized in a previously published study (19).

### Relative Risk

For each risk-outcome pair in the GBD study, the relative risk (RR) to the outcomes has been estimated as a function of exposure to risk factors. To achieve the RR estimation, the GBD study did meta-analyses of RRs from published systematic reviews in each GBD iterations. In GBD 2019, 81 new systematic reviews have been added. To estimate the RR for LBMD, twelve prospective observational studies reporting the RRs per standard deviation or per unit bone mass density were consulted in GBD 2017. In GBD 2019, only six of the 12 studies were used to extract the RR data, which were subsequently modeled using meta-regression-Bayesian, regularised, trimmed (MR-BRT), relaxing the log-linear assumption to allow for monotonically increasing or decreasing.

### Estimation of the Distribution of Risk Exposure by Age-Sex-Location-Year

To estimate the distribution of risk exposure, household surveys, censuses, published studies, and administrative data were searched for to estimate the mean levels of risk exposure. Then, the risk exposure data were modeled as a continuous parameter in the Bayesian meta-regression tool DisMod-MR 2.1 by age, sex, location and year (20, 21). In that model, age was split at 0, 10, 20, 30, 40, 50, 60, 70, 80, 90, and 100 years. The time window was set to 10 years for fitting data. The minimum coefficient of variation was 0.1 for global, 0.06 for super regions and 0.08 for other region level.

### Summary Exposure Value

In GBD 2019, the prevalence of risk factors was measured by the summary exposure value (SEV), which is weighted by the relative risk, taking the value zero indicating no excess risk for a population exists and the value one indicating the population is at the highest level of risk. In this study, the SEV represents the weighted prevalence of LBMD in the global and regional level. SEV ranges between 0 and 100 in the GBD 2019, with 100 indicating all the people are at maximum prevalence and 0 indicating all are at minimum prevalence. In this study, the reported SEV for LBMD was standardized by age. A decline in age-standardized SEV indicates decreased prevalence of LBMD, and vice versa.

### Theoretical Minimum-Risk Exposure Level for LBMD

The theoretical minimum risk level (TMREL) is defined as risk exposure level that minimizes the prevalence of risks at the population level. For LBMD, five cycles of the National Health and Nutrition Examination Survey I (NHANES) study were used as the reference population, and the TMREL was chosen as the age-sex specific 99th percentile of BMD.

### Socio-Demographic Index

The SDI is originally developed based on the methodology of the Human Development Index. It is a composite indicator of sociodemographic development status, which is strongly correlated with health outcomes. For a specific region, mean education for those 15 years old and older (EDU15+), the total fertility rate (TFR) for those younger than 25 years old (TFU25), and lag‐distributed income (LDI) per capita were used to calculated the SDI. It is the geometric mean of these three indices. An example to calculate SDI is shown in **Supplementary Material 3**. An SDI of 0 indicates that a country would have a theoretical minimum level of sociodemographic development related to the health outcomes, while a value of 1 indicates that a theoretical maximum level of sociodemographic development related to the health outcomes.

### Statistical Analysis

As LBMD is an age-related disease, the burden of LBMD was demonstrated as the age standardized rates (ASRs) of mortality (ASMR) and DALY (ASDR) by world standardized population. For a specific country, the ASR of mortality is calculated as $\sum_{k=1}^{A}\frac{n_{k}}{N_{k}}\beta_{k}*100000$ , where A is the total age groups, *n_k_* is the deaths associated with LBMD in age group *k*, *N_k_* is the total population in age group *k* in the country, and β_k_ is the proportion of the standardized population belonging to age group *k*. The estimated annual percentage change (EAPC) based on ASRs was used to reflect the temporal trends of SEV, ASMR and ASDR (22, 23). To calculate the EAPC, the age standardized SEV, ASMR, or ASDR was assumed to be linearly correlated with time, represented by the formula *y = α + βx + ϵ*. In this formula, y indicates log_10_ (ASR), while x is the calendar year. β is the regression coefficient of the linear model and the EAPC was calculated as EAPC = 100* (100 _β_ -1). The 95% confidence interval (95% CI) of EAPC can also be obtained by such calculation. If the EAPC value and its lower limit of 95% CI are above zero, the corresponding ASR was considered to have an upward trend, and vice versa. The correlation of SEV, ASMR and ASDR with SDI was analyzed by the Spearman rank order correlation. The detailed SDI for each country and territory was provided in **Supplementary Material 3**. P value less than 0.05 was considered to be statistically significant. For each estimate, we also demonstrated the 95% uncertainty interval (UI) to indicate the uncertainty of estimation. All statistical analyses and data visualization were performed in the R software (version 3.6.3).

## Results

### Global and Regional Prevalence of LBMD

The prevalence of LBMD is measured by the SEV as introduced above. **Table 1** shows the age standardized SEV of LBMD in 1990 and 2019 in the global and regional level. During this period, the global SEV for males, females and both sexes decreased [EAPC, -0.34 (95% CI, -0.38 ~ -0.3), -0.11 (95% CI, -0.13 ~ -0.08) and -0.2 (95% CI, -0.23 ~ -0.17), respectively]. In the regional level, the SEV declined for both sexes in all regions, except for high-income North America (EAPC, 0.2; 95% CI, 0.02 ~ 0.38). The highest decrease was seen in East Asia (EAPC, -0.64; 95% CI, -0.7 to ~ -0.57). The increase in high-income North America came from females (EAPC, 0.41; 95% CI, 0.25 ~ 0.58). In the other regions, the SEV decreased for both males and females, and the former showed greater decreases in most regions.

**Table 1:** Global and regional age-standardized SEV of low bone mineral density in 1990, 2019 and the temporal trends from 1990 to 2019 for males, females and both sexes.

Region	Both sexes				female				male		
	Age standardized SEV in 1990	Age standardized SEV in 2019	EAPC from 1990 to 2019		Age standardized SEV in 1990	Age standardized SEV in 2019	EAPC from 1990 to 2019		Age standardized SEV in 1990	Age standardized SEV in 2019	EAPC from 1990 to 2019
Global	17.1 (12.1 to 23.4)	16.3 (11.4 to 22.6)	-0.2 (-0.23 to -0.17)		21.1 (15.6 to 27.9)	20.7 (15 to 27.3)	-0.11 (-0.13 to -0.08)		12.3 (7.7 to 18.7)	11.3 (7 to 17.6)	-0.34 (-0.38 to -0.3)
High-middle SDI	15.8 (11 to 22)	15.2 (10.4 to 21.4)	-0.2 (-0.24 to -0.16)		19.7 (14.2 to 26.2)	19.3 (13.9 to 25.9)	-0.1 (-0.14 to -0.06)		10.9 (6.6 to 17)	10.3 (6.1 to 16.3)	-0.27 (-0.32 to -0.22)
High SDI	13.9 (9.4 to 20)	13.4 (8.9 to 19.5)	-0.13 (-0.19 to -0.06)		16.4 (11.6 to 22.7)	16.4 (11.4 to 22.8)	-0.01 (-0.07 to 0.06)		10.6 (6.4 to 16.7)	9.9 (5.8 to 16)	-0.19 (-0.28 to -0.1)
Low-middle SDI	19.1 (13.7 to 25.8)	17.9 (12.8 to 24.3)	-0.24 (-0.28 to -0.21)		24.4 (18.3 to 31.8)	22.8 (16.9 to 29.7)	-0.25 (-0.29 to -0.21)		13.8 (9 to 20.7)	12.6 (8 to 19)	-0.37 (-0.4 to -0.33)
Low SDI	21.8 (16.1 to 28.7)	20.7 (15 to 27.4)	-0.18 (-0.2 to -0.15)		27.6 (21.1 to 34.9)	26.2 (19.8 to 33.5)	-0.18 (-0.21 to -0.15)		16.1 (11 to 23)	15 (10 to 21.8)	-0.25 (-0.28 to -0.22)
Middle SDI	19.2 (13.9 to 25.7)	17.2 (12.3 to 23.6)	-0.44 (-0.48 to -0.4)		24.5 (18.6 to 31.2)	22.2 (16.5 to 28.9)	-0.38 (-0.41 to -0.35)		13.3 (8.4 to 20.1)	11.7 (7.2 to 18.2)	-0.56 (-0.62 to -0.51)
East Asia	20.7 (15.3 to 27.4)	18 (12.8 to 24.5)	-0.64 (-0.7 to -0.57)		26 (20 to 32.6)	22.9 (17.1 to 29.6)	-0.55 (-0.61 to -0.49)		14.4 (9.3 to 21.5)	12.4 (7.7 to 19.2)	-0.74 (-0.84 to -0.64)
Southeast asia	20.2 (15 to 26.9)	19.2 (14.1 to 25.6)	-0.17 (-0.2 to -0.15)		27 (21 to 34.1)	26 (19.9 to 32.8)	-0.13 (-0.16 to -0.11)		12.2 (7.5 to 18.6)	11.2 (6.7 to 17.6)	-0.32 (-0.35 to -0.29)
Oceania	15 (10.1 to 21.2)	14.7 (9.7 to 20.9)	-0.05 (-0.09 to -0.01)		20.7 (14.7 to 27.7)	20.2 (14.1 to 27.1)	-0.06 (-0.1 to -0.02)		9.4 (5.2 to 15.2)	9.3 (5.1 to 15.3)	0.01 (-0.04 to 0.07)
Central Asia	12.7 (8.2 to 18.4)	11.7 (7.4 to 17.6)	-0.2 (-0.24 to -0.16)		15.8 (10.6 to 22.3)	14.8 (9.7 to 21.4)	-0.15 (-0.19 to -0.11)		8.5 (4.7 to 14)	7.9 (4.2 to 13.1)	-0.23 (-0.27 to -0.18)
Central Europe	13.3 (8.9 to 19.2)	11.8 (7.6 to 17.5)	-0.38 (-0.42 to -0.35)		18.3 (12.9 to 25.3)	16.6 (11.3 to 23.2)	-0.32 (-0.34 to -0.29)		7.2 (3.7 to 12.2)	6.3 (3 to 11.1)	-0.43 (-0.49 to -0.38)
Eastern Europe	12.8 (8.5 to 18.7)	12.1 (8 to 17.8)	-0.19 (-0.23 to -0.16)		15.5 (10.5 to 21.7)	14.8 (9.9 to 20.9)	-0.18 (-0.22 to -0.15)		8.4 (4.7 to 13.8)	8.2 (4.6 to 13.6)	-0.03 (-0.08 to 0.01)
High-income Asia Pacific	17 (12 to 23.3)	14.7 (10.3 to 20.7)	-0.36 (-0.41 to -0.31)		21 (15.2 to 27.7)	18.7 (13.6 to 24.8)	-0.28 (-0.32 to -0.23)		12 (7.4 to 18.5)	10.3 (6.2 to 16.3)	-0.36 (-0.43 to -0.29)
Australasia	14.2 (9.7 to 20.5)	12.3 (7.6 to 18.5)	-0.52 (-0.55 to -0.48)		15.8 (11.5 to 22)	14 (8.9 to 20.7)	-0.44 (-0.46 to -0.41)		12 (7.2 to 18.9)	10.3 (5.6 to 16.5)	-0.54 (-0.61 to -0.48)
Western Europe	12.2 (8.2 to 18.2)	11.1 (7 to 17)	-0.35 (-0.39 to -0.31)		14.6 (10.2 to 20.7)	13.6 (8.9 to 19.8)	-0.28 (-0.31 to -0.25)		9.1 (5.2 to 14.9)	8.3 (4.5 to 14)	-0.32 (-0.37 to -0.26)
Southern Latin America	15.6 (10.7 to 22)	13.8 (9.1 to 20.1)	-0.44 (-0.47 to -0.41)		19.6 (13.8 to 26.9)	17.2 (11.8 to 24.1)	-0.46 (-0.48 to -0.43)		10.7 (6.1 to 17)	9.6 (5.4 to 15.7)	-0.39 (-0.43 to -0.35)
High-income North America	14 (9.4 to 20.4)	14.8 (9.9 to 21.3)	0.2 (0.02 to 0.38)		15.9 (11 to 22.3)	17.8 (12.5 to 24.7)	0.41 (0.25 to 0.58)		11.5 (7.1 to 17.9)	11.2 (6.8 to 17.8)	-0.04 (-0.27 to 0.2)
Caribbean	13.4 (9.1 to 19.7)	12 (7.5 to 18.1)	-0.44 (-0.47 to -0.41)		15.9 (11.1 to 22.1)	14.3 (9.3 to 20.4)	-0.43 (-0.46 to -0.4)		10.8 (6.5 to 17.1)	9.5 (5.2 to 15.5)	-0.48 (-0.51 to -0.45)
Andean Latin America	14.3 (9.3 to 20.8)	12.6 (8 to 18.7)	-0.46 (-0.49 to -0.43)		17.9 (12.2 to 25.1)	16 (10.4 to 22.7)	-0.43 (-0.46 to -0.4)		10.4 (5.9 to 16.9)	9 (5 to 15.1)	-0.55 (-0.59 to -0.52)
Central Latin America	16 (11 to 22.6)	14.7 (9.9 to 21)	-0.27 (-0.31 to -0.24)		21.4 (15.4 to 28.5)	19.8 (14 to 26.9)	-0.24 (-0.27 to -0.21)		10.3 (6 to 16.6)	8.9 (4.8 to 14.7)	-0.52 (-0.55 to -0.48)
Tropical Latin America	17.3 (12.1 to 24)	15.5 (10.3 to 22.2)	-0.4 (-0.43 to -0.37)		22.4 (16.5 to 29.7)	20.3 (14.1 to 27.6)	-0.35 (-0.38 to -0.32)		11.6 (7 to 18.2)	10 (5.7 to 16)	-0.57 (-0.61 to -0.53)
North Africa and Middle East	16.1 (11.3 to 22.5)	14.9 (10.1 to 21.2)	-0.24 (-0.27 to -0.21)		19.4 (14.1 to 25.9)	18 (12.8 to 24.5)	-0.24 (-0.26 to -0.21)		12.7 (8.2 to 19.4)	11.9 (7.4 to 18.2)	-0.22 (-0.25 to -0.19)
South Asia	18.2 (12.9 to 24.8)	17.1 (12 to 23.5)	-0.22 (-0.27 to -0.16)		23.6 (17.5 to 30.8)	22 (16.1 to 28.8)	-0.24 (-0.31 to -0.18)		13.4 (8.6 to 19.9)	12.1 (7.6 to 18.4)	-0.36 (-0.4 to -0.32)
Central Sub-Saharan Africa	23.8 (17.3 to 31.1)	23.2 (17.2 to 30.3)	-0.06 (-0.09 to -0.04)		28.9 (21.7 to 36.9)	28.5 (21.6 to 36.3)	-0.05 (-0.08 to -0.02)		17.9 (12.1 to 25.5)	17 (11.3 to 24.2)	-0.13 (-0.17 to -0.09)
Eastern Sub-Saharan Africa	24.9 (18.7 to 32.3)	23.6 (17.5 to 30.6)	-0.18 (-0.21 to -0.16)		30 (23.1 to 37.7)	28.6 (21.8 to 36.2)	-0.16 (-0.18 to -0.13)		19.6 (14 to 27)	18.1 (12.5 to 25.3)	-0.29 (-0.31 to -0.26)
Southern Sub-Saharan Africa	21.3 (15.4 to 28.5)	19.8 (14 to 26.8)	-0.23 (-0.27 to -0.19)		24.3 (17.9 to 31.5)	22.9 (16.6 to 30.1)	-0.18 (-0.22 to -0.14)		17.6 (11.9 to 24.8)	15.8 (10.4 to 22.6)	-0.38 (-0.42 to -0.33)
Western Sub-Saharan Africa	25.9 (19.7 to 33.1)	25.1 (18.9 to 32.5)	-0.07 (-0.1 to -0.05)		34.5 (27.3 to 42.4)	32.8 (25.7 to 40.7)	-0.16 (-0.18 to -0.14)		17.8 (12.3 to 24.8)	16.8 (11.3 to 23.7)	-0.19 (-0.23 to -0.16)

Data are presented in SEV with 95% UI. The SEV ranges from 0 to 100. SEV of 0 indicates that the total population is at minimum risk, while SEV of 100 indicates all the population is at aximum risk. For EAPC, data are presented in EAPC value with 95% confidence interval. SEV, summary exposure value; SDI, socio-demographic index; EAPC, estimated annual percentage change.

**Figure 1:**
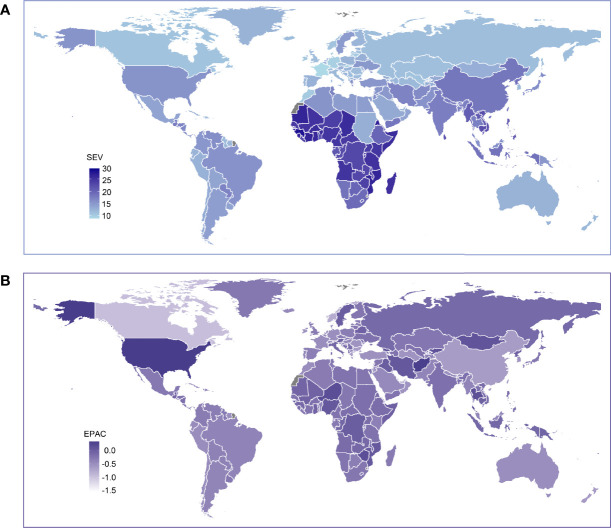
Global exposure to low bone mineral density. **(A)** Age standardized SEV of low bone mineral density, for both sexes in 204 countries and territories in 2019. **(B)** The EAPC in age standardized SEV of low bone mineral density, for both sexes from 1990 to 2019, in 204 countries and territories. SEV, summary exposure value; EAPC, estimated annual percentage change.

In the country level, high age standardized SEV in 2019 were mainly seen in countries located in eastern, central, and western Africa (**Figure 1A**). Togo had the highest SEV (29.2; 95% UI, 22.2-37.0), followed by Guinea and Eritrea. Countries in Western Europe had relatively low prevalence of LBMD, and France (8.9; 95% UI, 5.0-14.2) had the lowest prevalence among all countries and regions. The trends of age standardized SEV from 1990 to 2019 varied with countries. While most countries showed decreasing trends during this period, some countries had increasing prevalence with time, including the United States, Zimbabwe, Thailand, Georgia, Afghanistan, and Iran (**Figure 1B**). Among these countries, the United States had the highest increase of LBMD prevalence (EAPC, 0.32; 95% CI, 0.13 ~ 0.52), followed by Afghanistan (EAPC, 0.31; 95% CI, 0.20 ~ 0.41).

### Causes of LBMD-Related Mortality and Disability

For the 87 risk factors in GBD 2019, each risk factor is associated with an outcome or outcomes, defined as risk-outcome pairs (17). There are ten causes for LBMD -related mortality and disability (**Supplementary Figure 1**). In all years from 1990 to 2019, falls were associated with the highest ASMR as well as ASDR, followed by pedestrian road injuries and motor vehicle road injuries (**Supplementary Figure 1**). During this period, the ASMR associated with falls fluctuated around four deaths per 100,000, and the ASDR was over 120 DALYs per 100,000 in most years. There was a gradual decrease from 1990 to 2019 in the ASMR and ASDR for both pedestrian road injuries and Motor vehicle road injuries, while for other outcomes, the trends remained relatively stable.

### Global LBMD-Attributable Burden

Globally in 2019, there were about 438 thousand all-cause deaths and 16.6 million all-cause DALYs due to LBMD (**Supplementary Table 1**), increased by 111.1% and 93.8% respectively, compared to that in 1990. The ASMR and ASDR globally were 6 and 207 per 100,000, both of which decreased (ASMR, -0.22, 95% UI, -0.3 ~ -0.15; ASDR, 0.36, 95% UI, -0.4 ~ -0.33). In the regional level, high-income North America, Australia and East Asia had increased ASMR and ASDR, and the highest increase was seen in high-income North America for ASMR, and in Australia for ASDR (**Supplementary Table 1**). Other regions showed decreasing trends for both ASMR and ASDR.

**Figure 2:**
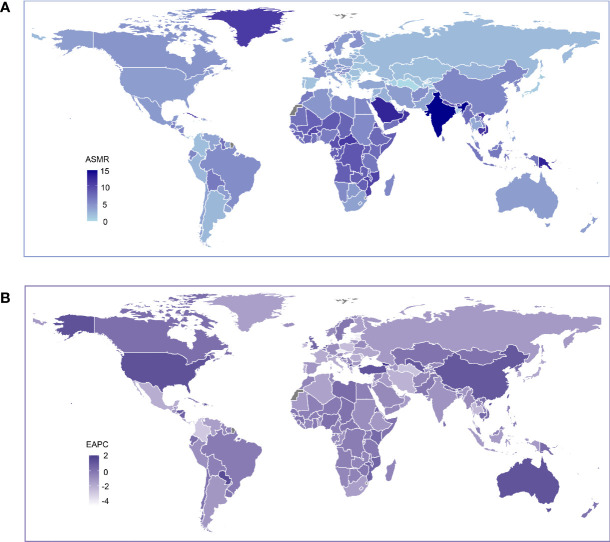
Global age standardized mortality rate to low bone mineral density. **(A)** The all-cause ASMR per 100,000 associated with low bone mineral density, for both sexes in 204 countries and territories in 2019. **(B)** The EAPC of ASMR of low bone mineral density, for both sexes from 1990 to 2019, in 204 countries and territories. ASMR, age standardized mortaility rate; EAPC, estimated annual percentage change.

**Figure 3:**
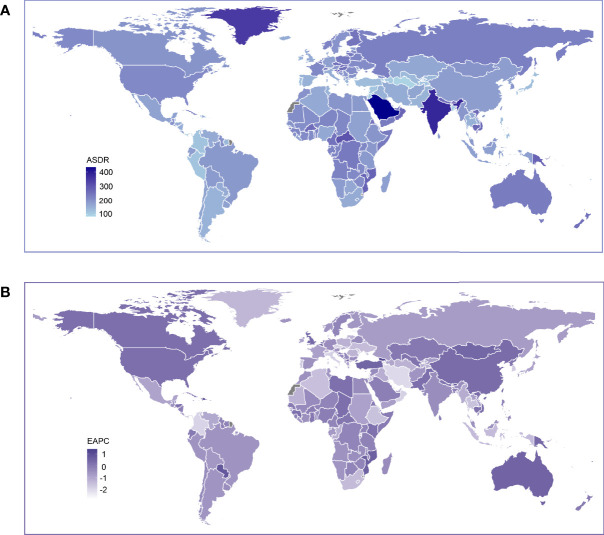
Global age standardized DALY rate of low bone mineral density. **(A)** The all-cause ASDR per 100,000 associated with low bone mineral density, for both sexes in 204 countries and territories in 2019. **(B)** The EAPC of ASDR of low bone mineral density, for both sexes from 1990 to 2019, in 204 countries and territories. DALY, disease adjusted life year. ASDR, age standardized DALY rate; EAPC, estimated annual percentage change.

In the country level, the ASMR in India, Solomon Islands, Papua New Guinea, Saudi Arabia, Bhutan, Vietnam, Cambodia, Oman, and Greenland exceeded ten deaths per 100,000, while Turkmenistan, Singapore, and Azerbaijan had a rate less than two deaths per 100,000 people (and **Figure 2A**). From 1990 to 2019, a decrease in LBMD attributable ASMR was observed in over 80% of all countries (**Figure 3B**). However, in some developed countries such as the United States, Australia, Canada, Netherlands, and in developing countries like China and Turkey, the ASMR increased with year (**Figure 2B**).

The ASDR in 2019 and its trends from 1990 to 2019 showed a similar geographical pattern to that of the ASMR (**Figure 3**). Increasing ASDR was seen in China, the United States, Canada, Australia and many other countries in Africa. Saudi Arabia (434 per 100,000) was the leading country in ASDR, followed by India (382 per 100,000) (**Supplementary Table 2**). The ASMR and ASDR in 2019 by sex are presented in **Supplementary Figures 2**–**5**. Generally, countries with relatively high mortality and DALY rates were mainly seen in the Middle East, South Asia and Central Africa.

### The Correlation of SEV and LBMD Attributable Burden to SDI

**Figure 4:**
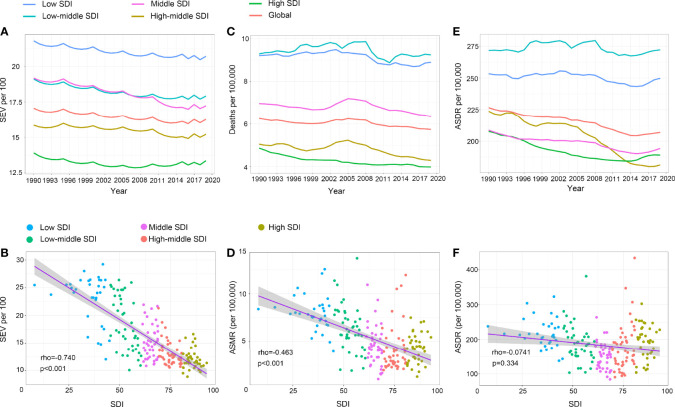
The exposure and burden of LBMD by SDI. **(A, C, E)** The age standardized SEV, ASMR and ASDR of LBMD in different SDI regions from 1990 to 2019. Results are showed for both sexes worldwide. **(B, D, F)** The correlation between SEV and SDI **(B)**, ASMR and SDI **(D)**, ASDR and SDI **(F)**. Rho indicates the value of the Spearman rank order correlation tests. SEV, summary exposure value; ASMR, age standardized mortaility rate; DALY, disease adjusted life year. ASDR, age standardized DALY rate.

**Figure 5:**
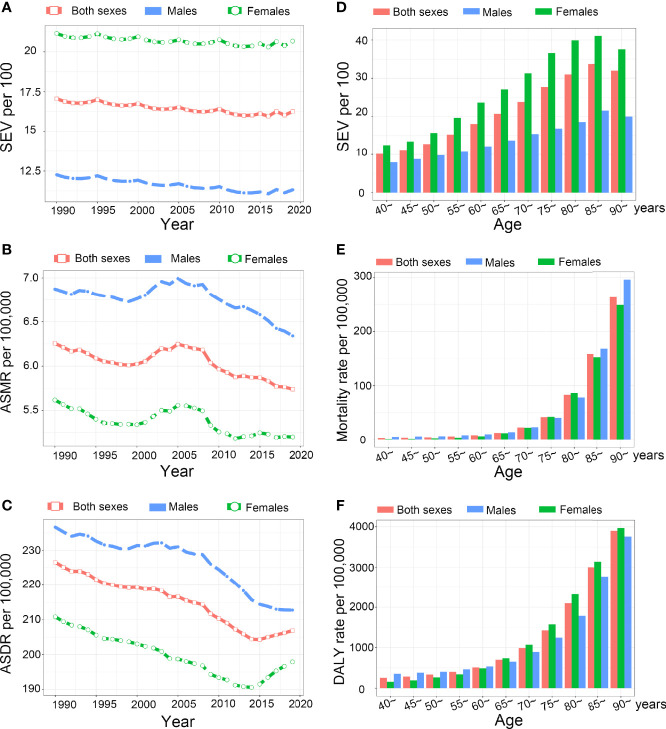
The exposure and burden of LBMD by age and sex. **(A-C)** The all-cause age standardized SEV, ASMR and ASDR of LBMD worldwide from 1990 to 2019. **(D-F)** The SEV **(D)**, mortality rate **(E)**, and disability rate **(F)** in different age groups. SEV, summary exposure value; ASMR, age standardized mortaility rate; DALY, disease adjusted life year. ASDR, age standardized DALY rate.

Since 1990, the age standardized SEV gradually decreased in the global level and in all SDI regions, but high SDI regions demonstrated a slightly reversing trend since 2008 (**Figure 4A**). During this period, low SDI was associated with the highest SEV, while high SDI was associated with the lowest SEV in all years. Spearman’s rank correlation coefficient revealed a strong negative association between age standardized SEV and SDI levels (**Figure 4B**, rho = -0.74, p < 0.001) in 2019. For the LBMD attributable burden, while the ASMR showed a decreasing trend with increasing SDI levels, there was no obvious correlation between the ASDR and SDI (**Figures 4C–F**). Unlike the SEV of LBMD, low-middle SDI regions ranked top in the mortality and DALY rate attributable to LBMD in all years from 1990 to 2019 (**Figures 4C, E**). High SDI regions had the lowest ASDR in most years during this period, but the trend gradually increased since 2014, which was also the same in low, low-middle, middle SDI regions, as well as in the global level (**Figure 5E**). This ascending DALY rate was mainly attributed to the increase in female people, because the trend remained stable or even decreased in males in most SDI regions (**Supplementary Figure 6**).

### SEV and Low-BMD Attributable Burden by Sex and Age

Females have higher prevalence to LBMD than males in all years from 1990 to 2019 (**Figure 5A**). However, the ASMR and ASDR were lower in females during this period (**Figures 5B, C**). As LBMD mostly affects elderly people, the data regarding LBMD are available only for people aged 40 years or older in GBD 2019. The SEV in 2019 was the lowest at the 40-45 age group. Then, it increased with age and peaked at the 85 to 89-year group. Females had higher SEVs in all age groups and the increasing speed was faster with age than that of males (**Figure 5D**). The mortality rate and DALY rate also increased with age and peaked in the 90+ year group. Before 65 years of age, the mortality and DALY rates were lower in females, but the DALY rate in females exceeded that in males after 65 years of age (**Figures 5E, F**).

## Discussion

In this study, we found he global prevalence of LBMD has decreased in the past thirty years, and most countries have decreasing trends of LBMD attributable burden. However, some countries, including many high-income countries, have increasing burden from 1990 to 2019. The SEV and ASMR are negatively correlated with SDI. LBMD is more common in females, but the attributable disability and mortality are higher in males in all years from 1990 to 2019.

The decreasing prevalence of LBMD from 1990 to 2019 in almost all regions might be a result of the progress in controlling LBMD worldwide. However, it should be noted that the population worldwide has increased at a high speed (19). It’s possible that as the increased population become old, the world will be more burdened by LBMD and osteoporosis. In addition, we observed a negative correlation between the SEV of LBMD and and SDI, indicating that people in low-income countries are more prone to LBMD than developed countries. Such correlation may be explained by ethnicity background, anthropometric variables, and nutrition status (24). Deficient nutrition intake, such as calcium and high-quality protein, has been proved to predict lower bone mass (25). However, in low SDI regions, people tend to have less access to adequate nutrition supply, including milk, cheese, and yoghurt, which are associated with better bone health (26). It is estimated that about 40% to 60% of total bone mass in the adulthood is gained during adolescence, so it is important to guarantee sufficient nutrients intake, such as high-quality protein, calcium, and vitamin D in the adolescents to promise higher bone mass in their future life (26). In addition to the prevention of LBMD, the treatment of osteoporosis also remains a big challenge in low-SDI countries, because the mortality and disability rate were also higher in these countries.

Although the SEV to LBMD decreased in most SDI regions from 1990 to 2019, the gradual increase since 2008 in high SDI regions is noteworthy. High-income North America even had increasing prevalence of LBMD from 1990 to 2019. Reasons for such results are complicated. Prolonged life expectancy and increased proportion of elderly people in highly developed countries may have a role, as LBMD is an age-related condition affecting mainly elderly people. Underuse of bone protective drugs may be another important reason. In the United Kingdom, the yearly rate of anti-osteoporotic drug prescription has reached a plateau since 2006, compared to an ever-ageing population (27). In the USA, the use of such drugs even decreased from 2001 to 2011 (28). As a result, osteoporosis might be undertreated in these countries.

Osteoporosis predisposes people to high risk of falls, which were the most common outcome attributable to LBMD in this study. Falls may be accounted for by hip fractures, one type of the two typical osteoporotic fractures (29). Falls in return can result in hip fractures due to mechanical force upon falling, leading to substantial disability, increased risk of mortality, and increased medical cost (29, 30). From 2010 to 2019, there was a global decrease in ASMR and ASDR of LBMD. However, the situation of LBMD prevention and control varied with countries. As most countries had decreased LBMD attributable burden, upward trends were seen in many countries, even in highly developed countries, such as the United States, Australia, Canada, and Netherlands. In addition, it should also be noted that during the past decades, although the rate declined worldwide, the absolute number of deaths and DALYs attributable to LBMD increased in almost all the countries, along with the increasing population and the process of aging worldwide. These will definitely add to the medical and economic burden to the society, and greater challenges will come if efforts are not fully taken to deal with LBMD and osteoporosis in the population.

Female people are more likely to suffer from LBMD and osteoporosis due to the protective effects of estrogen on bone after menopause. Globally, the prevalence of LBMD was higher in females in all years from 1990 to 2019. However, after adjusting for age, LBMD-attributable disability and mortality rate were higher in males. There is a common misconception that LBMD and osteoporosis affects only post-menopause women. Indeed, osteoporosis in men has been overlooked for too long (5). Underdiagnosis and undertreatment of osteoporosis may underline the lower prevalence but higher mortality and disability rate in men for the past decades. It is of great importance that clinicians gain awareness of detecting and treating osteoporosis in men. LBMD principally affects old people, and the prevalence and attributable burden increased with age. A sex-difference was seen in the SEV of LBMD and the attributable burden. Females had higher SEV in all years from 1990 to 2019 and in people older than 40 years. After menopause, females are more prone to bone loss due to the lack of protective effects from estrogen on the skeletal system, and additional care should be taken on skeletal health for old female people.

Severe adverse events due to LBMD can be prevented in people at high risk by standard treatment. Bisphosphonates play an essential role in the management of osteoporosis during the past decades. However, poor adherence to bisphosphonate therapy, fear of adverse effects and economic burden from long-term use have resulted in inadequate use of anti-osteoporotic drugs and undertreatment of osteoporosis (29, 31). Severe adverse effects of bisphosphonate therapy include atypical femoral fractures and osteonecrosis of the jaw (32), which, however, occur very rarely and can be reduced by proper preventive measures (33). The majority of patients can benefit from proper treatment with anti-resorptive agents. Teriparatide and the RANK ligand inhibitor denosumab are more effective in preventing fractures than bisphosphonates, but the incremental cost of these drugs is the main obstacle to extensive use, especially in low-income countries (34). In addition to drug therapy, increasing health care workers’ awareness of osteoporosis treatment, enhancing medical consulting to patients about the use of drugs and regimes, promoting public education on the harm of osteoporosis, and supportive policies will also take effect to alleviate the severe situation of LBMD.

An earlier study evaluating the prevalence and burden of LBMD in the global scale was conducted in 2014 using data from the GBD study 2010 (35). In GBD 2019, the technical quantification of LBMD attributable burden has been advanced by including more countries and larger sample size, as well as by improving the evaluating methods. Using data from the GBD 2019, we also reported the trends in the prevalence and burden of LBMD in recent years, thus providing a comprehensive understanding about the current situation. However, this study also has some limitations. As the mean BMD was not reported for all samples in specific age and sex groups when inputting the data, the total mean BMD at the population level was represented by aggregated mean BMD of available data in specific groups and potential bias may exist. Another factor affecting the accuracy of estimation is the wide range of 95% UIs in some countries and regions, which may reduce the power to detect the discrepancies among different countries, years, sex, or age groups. In addition, people in low SDI regions will have lower access to screening programs and treatment of osteoporosis, which may lead to inadequate detection of osteoporosis in the population, leading to potential bias in estimating the actual prevalence and burden of LBMD in low SDI countries. More accurate estimation will be made if more primary data are obtained in future rounds of GBD studies.

## Conclusion

Decreasing trends of worldwide prevalence of LBMD and the associated burden have been seen from 1990 to 2019. However, some countries, including many high-income countries, have increasing trends of prevalence, mortality and disability rate from LBMD. Compared to high-income countries, low-income countries have higher LBMD prevalence and face more burden from LBMD. The treatment of osteoporosis have been overlooked in men for long time. With population aging worldwide, we will face increasing challenges and effective measures in the healthcare worker, the public and policy level are warranted to control the burden of LBMD.

## Data Availability Statement

The datasets generated for this study can be found in the the website of the GBD study 2019 [http://ghdx.healthdata.org/gbd-results-tool]. Further inquiries can be directed to the corresponding authors.

## Author Contributions

Conceptualization, design: FL. Data collection and curation: DWY. Drafting of the manuscript and software analysis: YMD. Critical revision: HLK, RPP, KHS, QG, MPZ. Supervision: HFG. FL and DWY contributed equally as co-corresponding authors. All authors contributed to the article and approved the submitted version.

## Funding

This project was supported by grants from the National Natural Science Foundation of China (82072500).

## Conflict of Interest

The authors declare that the research was conducted in the absence of any commercial or financial relationships that could be construed as a potential conflict of interest.

## Publisher’s Note

All claims expressed in this article are solely those of the authors and do not necessarily represent those of their affiliated organizations, or those of the publisher, the editors and the reviewers. Any product that may be evaluated in this article, or claim that may be made by its manufacturer, is not guaranteed or endorsed by the publisher.

## Supplementary Material

Supplementary Figure 1 The top ten causes for LBMD-related outcomes by ASMR and ASDR worldwide for both sexes from 1990 to 2019.

Supplementary Figure 2 Global age standardized mortality rate to low bone mineral density for females in 204 countries and territories in 2019.

Supplementary Figure 3 Global age standardized mortality rate (ASMR) per 100,000 to low bone mineral density for males in 204 countries and territories in 2019.

Supplementary Figure 4 Global age standardized DALY rate (ASDR) per 100,000 to low bone mineral density for females in 204 countries and territories in 2019.

Supplementary Figure 5 Global age standardized DALY rate (ASDR) per 100,000 to low bone mineral density for males in 204 countries and territories in 2019.

Supplementary Figure 6 The age standardized DALY rate (ASDR) per 100,00 of LBMD for male **(A)** and female **(B)** in different SDI regions from 1990 to 2019.

## Abbreviations

LBMD, low bone mineral density; DALY, disability-adjusted life years; SEV, summary exposure value. SDI, socio-demographic index; GBD, the Global Burden of Disease Study; CRA, comparative risk assessment; ASMR, age standardized mortality rate; ASDR, age standardized DALY rate; UI, uncertainty interval; EAPC, estimated annual percentage change.
