# Supplementary figures and images for: Global, Regional, and National Burden of Low Bone Mineral Density From 1990 to 2019: Results From the Global Burden of Disease Study 2019
Source: Front Endocrinol (Lausanne). 2022 May 24;13:870905. doi: 10.3389/fendo.2022.870905 (PMC9172621; doi:10.3389/fendo.2022.870905)

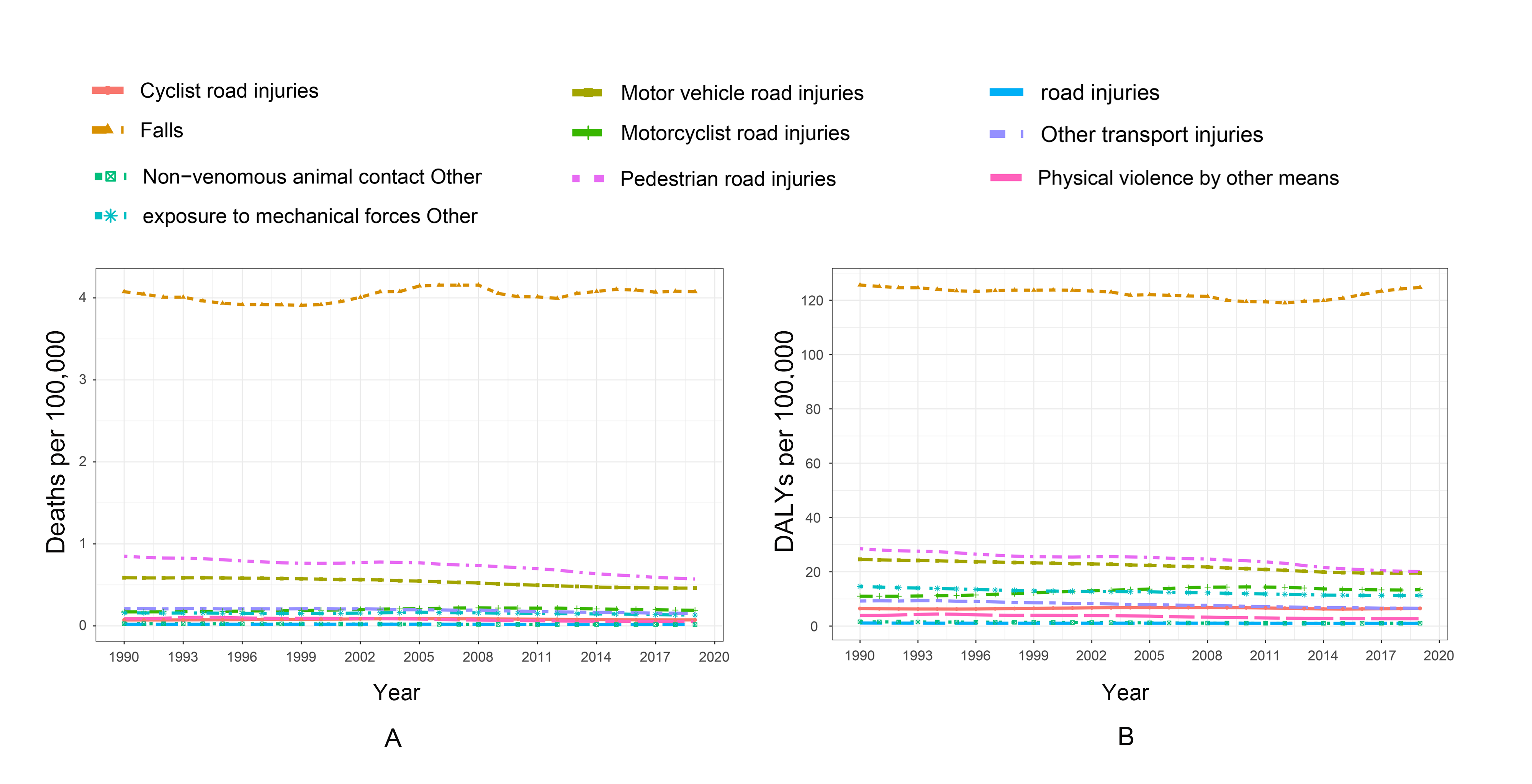

Supplement: Supplementary Figure 1 — The top ten causes for LBMD-related outcomes by ASMR and ASDR worldwide for both sexes from 1990 to 2019. [file Image_1.tif]

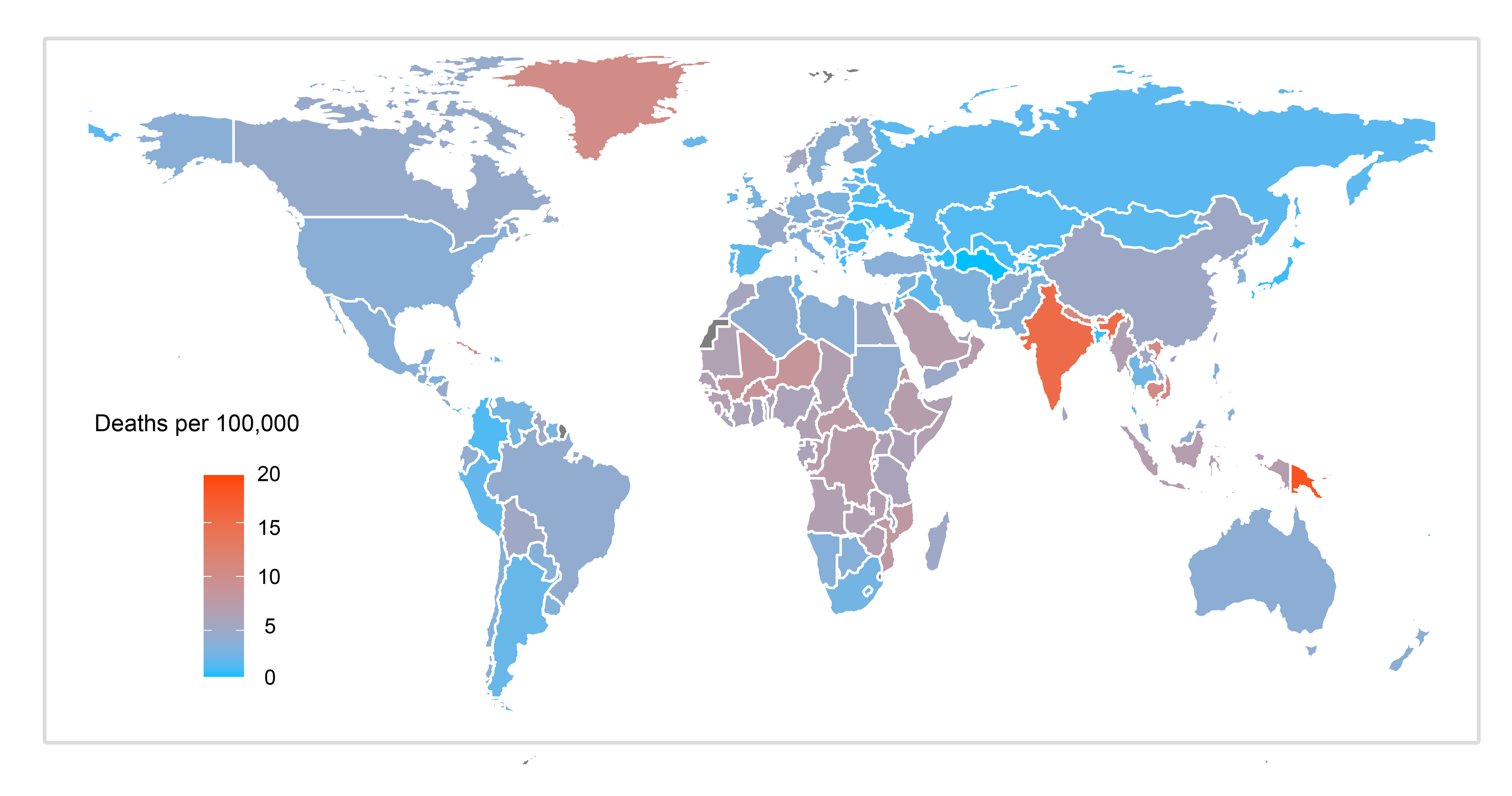

Supplement: Supplementary Figure 2 — Global age standardized mortality rate to low bone mineral density for females in 204 countries and territories in 2019. [file Image_2.tif]

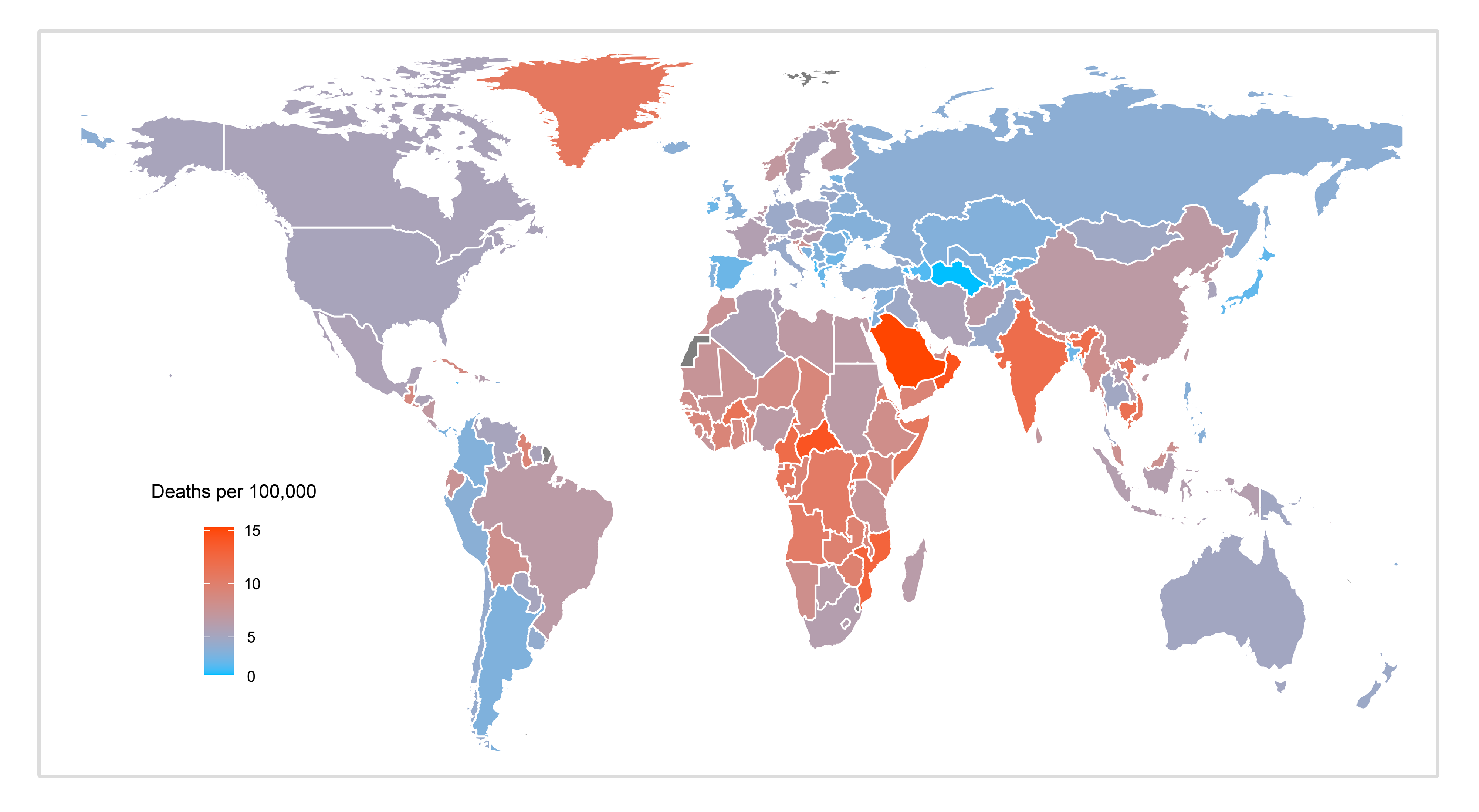

Supplement: Supplementary Figure 3 — Global age standardized mortality rate (ASMR) per 100,000 to low bone mineral density for males in 204 countries and territories in 2019. [file Image_3.tif]

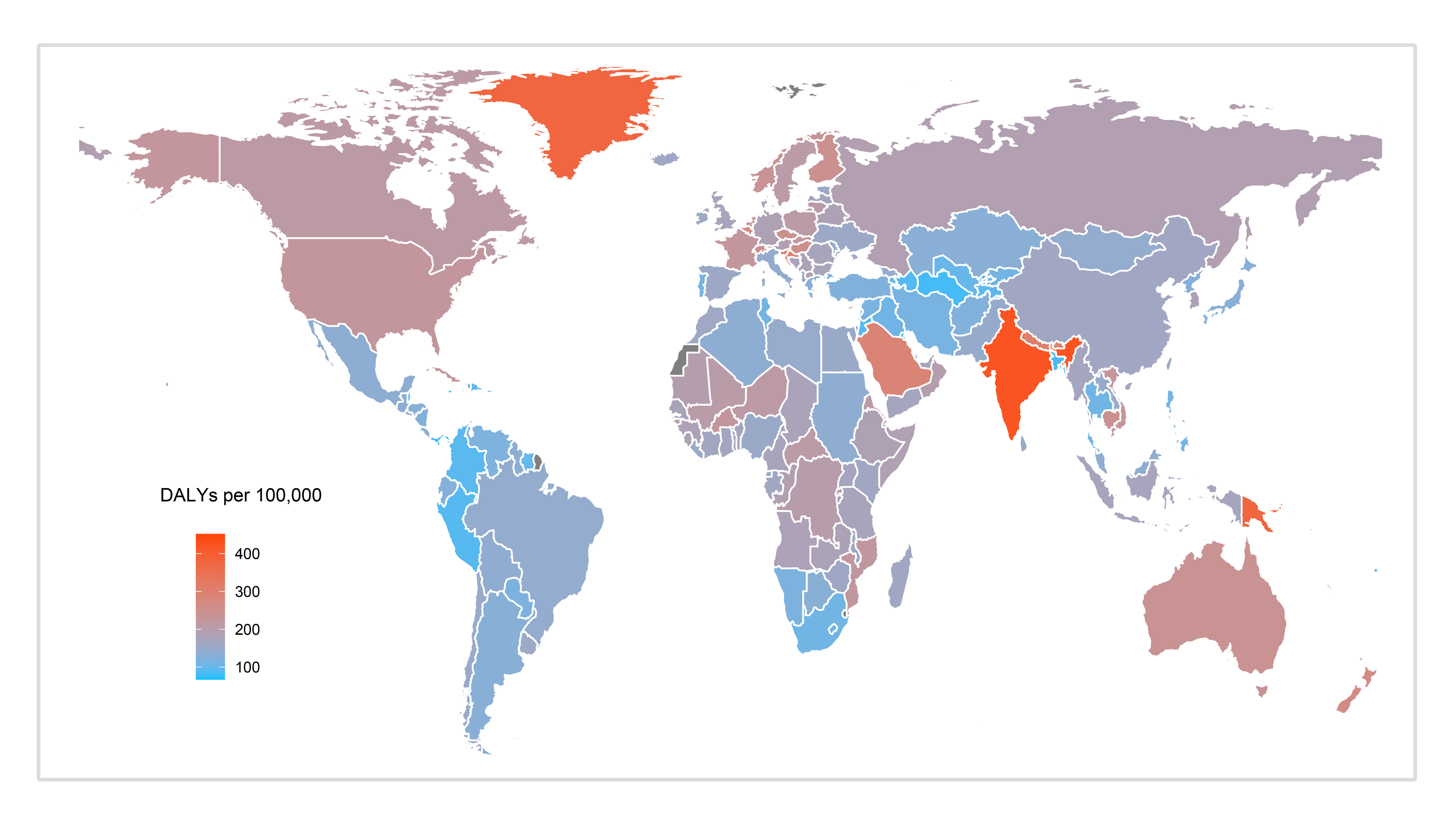

Supplement: Supplementary Figure 4 — Global age standardized DALY rate (ASDR) per 100,000 to low bone mineral density for females in 204 countries and territories in 2019. [file Image_4.tif]

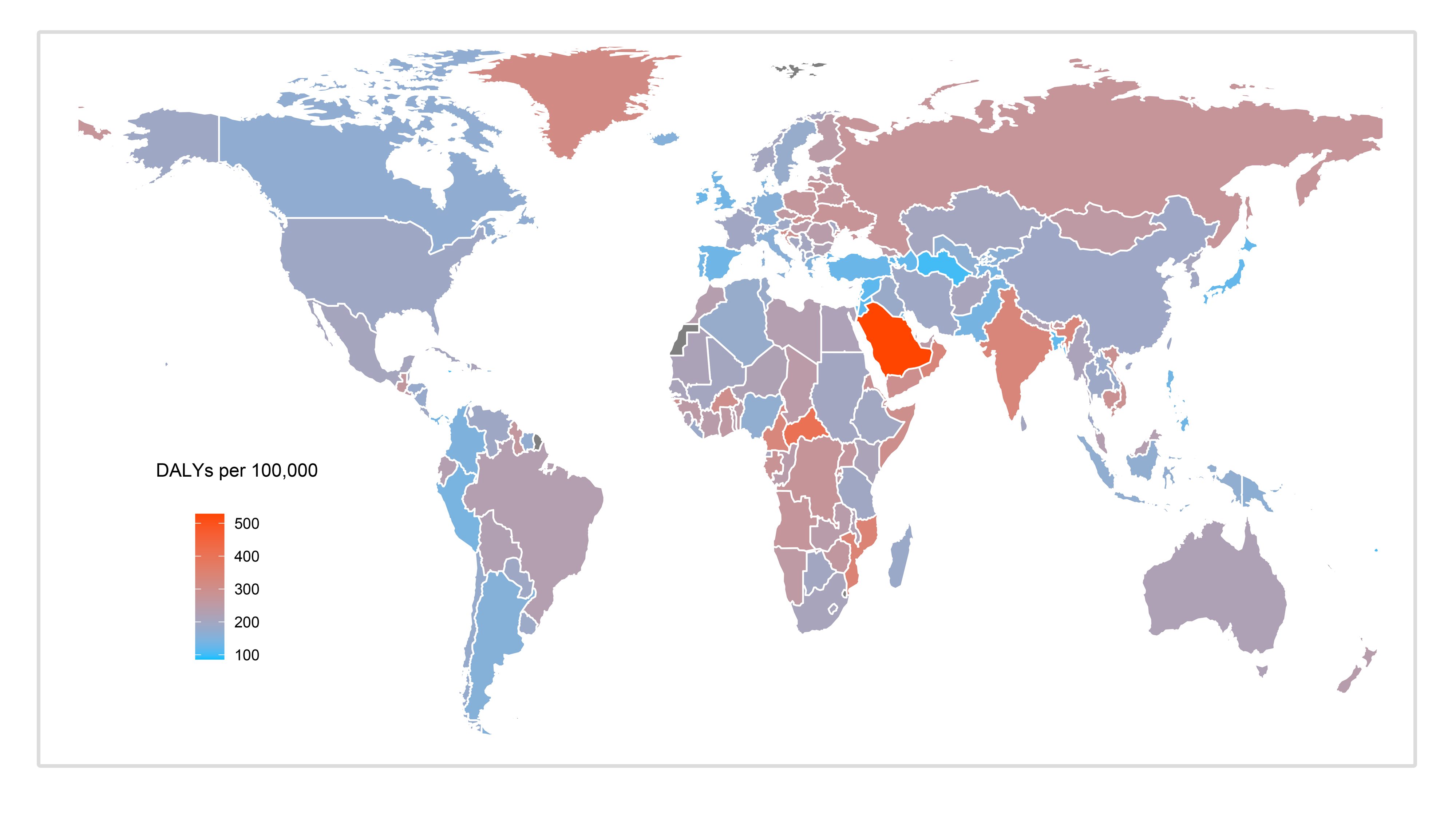

Supplement: Supplementary Figure 5 — Global age standardized DALY rate (ASDR) per 100,000 to low bone mineral density for males in 204 countries and territories in 2019. [file Image_5.tif]

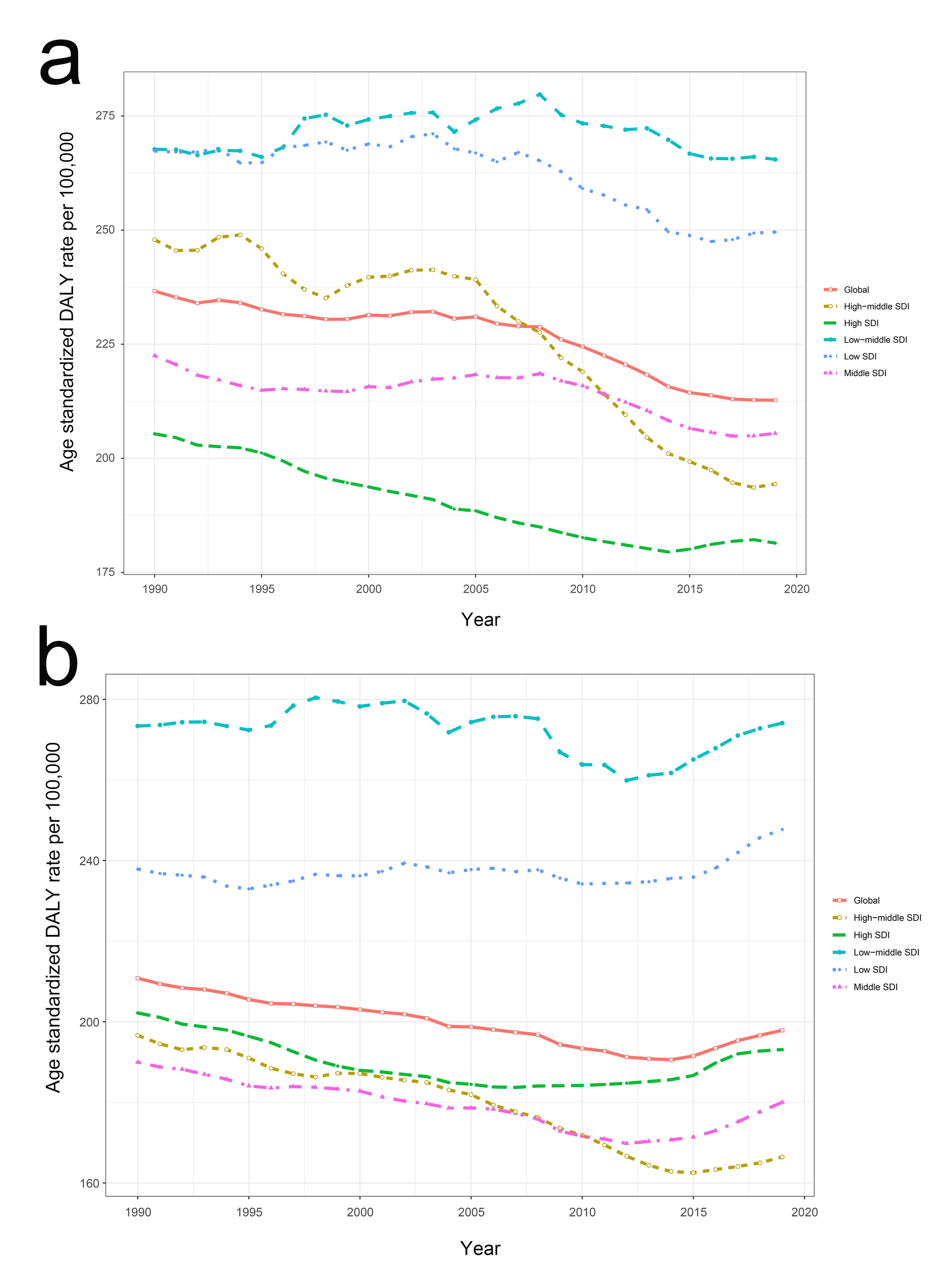

Supplement: Supplementary Figure 6 — The age standardized DALY rate (ASDR) per 100,00 of LBMD for male (A) and female (B) in different SDI regions from 1990 to 2019. [file Image_6.tif]
