# Supplementary material for: Global, Regional, and National Burden of Low Bone Mineral Density From 1990 to 2019: Results From the Global Burden of Disease Study 2019
Source: Front Endocrinol (Lausanne). 2022 May 24;13:870905. doi: 10.3389/fendo.2022.870905 (PMC9172621; doi:10.3389/fendo.2022.870905)
Supplement: Supplementary file 7 [file Table_1.docx]

**Supplementary table 1.** the mortality and DALY associated with low bone mineral density in 1990 and 2019 in worldwide and in 21 regions.

| location | death number in 1990 (95% UI) | death numner in 2019 (95% UI) | ASMR in 2019 (95% UI) | EAPC of ASMR from 1990 to 2019 (95% CI) | DALY number 2019 (95% UI) | DALY number 2019 (95% UI) | ASDR 2019 (95% UI) | EAPC of ASDR from 1990 to 2019 (95% CI) |
| --- | --- | --- | --- | --- | --- | --- | --- | --- |
| Global | 207,367 (179,508 to 226,355) | 437,884 (361,105 to 495,521) | 6 (5 to 7) | -0.22 (-0.3 to -0.15) | 8,588,936 (7,041,725 to 10,138,619) | 16,647,466 (13,503,526 to 20,036,302) | 207 (168 to 249) | -0.36 (-0.4 to -0.33) |
| East Asia | 33,686 (27,709 to 44,462) | 92,940 (61,782 to 113,617) | 6 (4 to 7) | 0.92 (0.53 to 1.31) | 1,402,729 (1,157,004 to 1,708,995) | 3,418,853 (2,689,793 to 4,113,484) | 175 (137 to 211) | 0.24 (0.14 to 0.34) |
| Southeast asia | 16,276 (11,751 to 18,886) | 31,114 (23,085 to 35,981) | 6 (4 to 7) | -1.06 (-1.14 to -0.98) | 551,067 (452,128 to 634,490) | 1,046,320 (856,651 to 1,209,786) | 178 (146 to 206) | -0.95 (-1.01 to -0.88) |
| Oceania | 170 (103 to 219) | 450 (227 to 624) | 10 (4 to 14) | 0.15 (-0.11 to 0.42) | 6,035 (4,523 to 7,307) | 15,736 (11,191 to 19,817) | 235 (152 to 302) | 0.25 (0.07 to 0.42) |
| Central Asia | 1,194 (992 to 1,311) | 1,553 (1,267 to 1,774) | 2 (2 to 3) | -0.07 (-0.36 to 0.23) | 75,629 (59,044 to 92,198) | 107,835 (82,693 to 133,742) | 139 (107 to 173) | -0.27 (-0.45 to -0.1) |
| Central Europe | 9,581 (8,085 to 10,520) | 8,338 (6727 to 9,723) | 4 (3 to 5) | -2.76 (-2.89 to -2.64) | 455,482 (356,435 to 565,218) | 475,447 (359,193 to 610,371) | 231 (174 to 298) | -1.35 (-1.44 to -1.26) |
| Eastern Europe | 8,703 (7,301 to 9,588) | 8,648 (7,021 to 10,056) | 3 (2 to 3) | -1.46 (-1.95 to -0.97) | 733,463 (560,615 to 930,248) | 722,830 (545,173 to 938,839) | 221 (167 to 287) | -0.97 (-1.26 to -0.68) |
| High-income Asia Pacific | 6,192 (5,317 to 6,695) | 12,681 (9,738 to 14,651) | 2 (2 to 3) | -1.83 (-1.95 to -1.7) | 354,428 (281,174 to 443,904) | 621,696 (480,014 to 804,733) | 139 (107 to 182) | -1.09 (-1.17 to -1) |
| Australasia | 843 (720 to 932) | 2,565 (1,995 to 2,973) | 4 (3 to 5) | 0.96 (0.77 to 1.15) | 50,381 (38,634 to 65,767) | 118,366 (90,435 to 154,609) | 235 (178 to 309) | 0.38 (0.3 to 0.45) |
| Western Europe | 33,070 (27,381 to 36,926) | 46,194 (36,415 to 52,825) | 4 (3 to 5) | -1.24 (-1.4 to -1.07) | 1,173,323 (926,658 to 1,459,168) | 1,669,664 (1,284,507 to 2,129,111) | 174 (134 to 223) | -0.71 (-0.79 to -0.63) |
| Southern Latin America | 1,726 (1,478 to 1,874) | 2,776 (2,304 to 3,094) | 3 (3 to 4) | -0.79 (-0.89 to -0.69) | 78,702 (63,220 to 95,839) | 124,988 (97,849 to 156,480) | 152 (119 to 191) | -0.54 (-0.58 to -0.5) |
| High-income North America | 12,260 (10,410 to 13,450) | 31,653 (26,113 to 35,362) | 5 (4 to 5) | 1.18 (1.02 to 1.35) | 663,879 (520,119 to 838,747) | 1,310,719 (1,033,102 to 1,645,337) | 209 (165 to 262) | 0.22 (0.14 to 0.31) |
| Caribbean | 1397 (1,176 to 1,566) | 3,222 (2,557 to 3,830) | 6 (5 to 7) | -0.27 (-0.45 to -0.09) | 43,337 (35,990 to 50,357) | 88,226 (71,295 to 104,822) | 170 (137 to 202) | -0.19 (-0.33 to -0.06) |
| Andean Latin America | 986 (825 to 1,130) | 2,278 (1,777 to 2,784) | 4 (3 to 5) | -0.54 (-0.64 to -0.44) | 35,346 (29,117 to 40,432) | 80,330 (63,236 to 96,245) | 141 (111 to 169) | -0.44 (-0.52 to -0.36) |
| Central Latin America | 4,954 (4,244 to 5,354) | 9,272 (7,618 to 10,911) | 4 (3 to 5) | -1.96 (-2.16 to -1.77) | 193,051 (160,231 to 222,883) | 368,835 (297,303 to 437,749) | 155 (125 to 184) | -1.12 (-1.29 to -0.96) |
| Tropical Latin America | 4,988 (4,294 to 5,387) | 12,377 (10,216 to 13,856) | 5 (4 to 6) | -0.24 (-0.48 to 0) | 211,770 (177,059 to 242,641) | 447,687 (366,911 to 523,952) | 185 (152 to 217) | -0.57 (-0.68 to -0.45) |
| North Africa and Middle East | 9,898 (7,554 to 11,324) | 20,371 (14,848 to 24,374) | 5 (4 to 6) | -0.65 (-0.74 to -0.57) | 377,386 (297,022 to 436,564) | 805,959 (630,238 to 959,581) | 175 (137 to 208) | -0.64 (-0.66 to -0.61) |
| South Asia | 48,166 (38,980 to 55,639) | 125,838 (101,842 to 148,032) | 12 (9 to 14) | -0.53 (-0.74 to -0.31) | 1,725,813 (1,430,996 to 2,002,773) | 4,339,280 (3,545,372 to 5,185,159) | 335 (276 to 398) | -0.18 (-0.3 to -0.06) |
| Central Sub-Saharan Africa | 1,702 (1,366 to 2,334) | 3,800 (2,893 to 5,136) | 9 (7 to 11) | -0.41 (-0.46 to -0.36) | 59,250 (47,899 to 77,044) | 132,808 (102,466 to 171,974) | 236 (186 to 302) | -0.37 (-0.42 to -0.33) |
| Eastern Sub-Saharan Africa | 4,845 (4,160 to 5,476) | 9,192 (7,914 to 10,320) | 8 (7 to 9) | -0.48 (-0.52 to -0.44) | 167,585 (143,454 to 191,642) | 313,649 (265,084 to 359,114) | 203 (173 to 231) | -0.6 (-0.64 to -0.56) |
| Southern Sub-Saharan Africa | 1,663 (1,380 to 1,849) | 2,521 (2,157 to 2,829) | 5 (4 to 5) | -1.2 (-1.57 to -0.83) | 65,331 (54,001 to 73,678) | 98,304 (82,322 to 111,029) | 164 (138 to 186) | -1.15 (-1.4 to -0.89) |
| Western Sub-Saharan Africa | 5,070 (4,268 to 5,953) | 10,102 (8,397 to 11,992) | 7 (6 to 9) | -0.11 (-0.19 to -0.04) | 164,948 (139,222 to 193,177) | 339,935 (283,468 to 396,771) | 190 (160 to 221) | -0.13 (-0.16 to -0.1) |

Abbreviations: UI, uncertainty interval; CI, confidence interval; ASMR, age standardized mortality rate; ASDR, , age standardized DALY rate
