# Supplementary material for: Global, Regional, and National Burden of Low Bone Mineral Density From 1990 to 2019: Results From the Global Burden of Disease Study 2019
Source: Front Endocrinol (Lausanne). 2022 May 24;13:870905. doi: 10.3389/fendo.2022.870905 (PMC9172621; doi:10.3389/fendo.2022.870905)
Supplement: Supplementary file 8 [file Table_2.docx]

**Supplementary table 2.** Low bone mineral density associated burden in 2019 and the estimated annual percentage from 1990 to 2019 by country.

| location | Number of deaths in 2019 | ASMR in 2019 (per 100,000) | EAPC of ASMR from 1990 to 2019 | Number of DALYs in 2019 (in thousands) | ASDR in 2019 (per 100,000) | EAPC of ASDR from 1990 to 2019 |
| --- | --- | --- | --- | --- | --- | --- |
| Global | 437,884 (361,105 to 495,521) | 5.75 (4.72 to 6.51) | -0.22 (-0.3 to -0.15) | 16,647.5 (13,503.5 to 20,036.3) | 206.85 (167.92 to 248.69) | -0.36 (-0.40 to -0.33) |
| Global males | 209,587 (173,630 to 236,460) | 6.34 (5.30 to 7.15) | -0.19 (-0.27 to -0.11) | 7,990.9 (6,480.0 to 9,429.6) | 212.73 (173.10 to 250.89) | -0.36 (-0.42 to -0.31) |
| Global females | 228,298 (177,697 to 266,439) | 5.20 (4.05 to 6.07) | -0.21 (-0.29 to -0.13) | 8,656.6 (6935.4 to 10,586.1) | 197.89 (158.48 to 242.01) | -0.31 (-0.38 to -0.26) |
| China | 89,857 (58,927 to 110,423) | 5.78 (3.58 to 7.16) | 1.01 (0.61 to 1.41) | 3,320.28 (2,594.48 to 4,004.26) | 177.21 (138.17 to 214.21) | 0.3 (0.19 to 0.41) |
| North Korea | 1,662 (1,284 to 2,082) | 5.67 (4.37 to 7.01) | 0.39 (0.16 to 0.62) | 53.99 (42.17 to 67.71) | 165.96 (130.2 to 206.76) | 0.11 (0 to 0.21) |
| Taiwan (Province of China) | 1,421 (1,084 to 1,825) | 3.55 (2.7 to 4.57) | -2.22 (-2.48 to -1.96) | 44.58 (35.18 to 55.27) | 113.73 (89.64 to 140.97) | -2.54 (-2.76 to -2.33) |
| Cambodia | 961 (765 to 1,148) | 10.93 (8.69 to 12.95) | 0.36 (0.28 to 0.44) | 29.1 (23.97 to 34.41) | 268.22 (222.33 to 314.2) | 0.19 (0.11 to 0.27) |
| Indonesia | 10,053 (8,106 to 11,591) | 6.69 (5.09 to 7.81) | -1.39 (-1.66 to -1.13) | 349.9 (285.96 to 406.52) | 174.71 (144.75 to 202.86) | -1.45 (-1.64 to -1.25) |
| Laos | 196 (156 to 235) | 5.68 (4.6 to 6.76) | -0.57 (-0.62 to -0.51) | 7.18 (5.75 to 8.6) | 165.6 (133.8 to 195.69) | -0.83 (-0.89 to -0.77) |
| Malaysia | 1,400 (1,024 to 1,801) | 6.16 (4.43 to 7.86) | -0.56 (-0.75 to -0.37) | 48.7 (38.18 to 59.64) | 186.37 (146.04 to 227.58) | -0.52 (-0.61 to -0.43) |
| Maldives | 8 (7 to 10) | 3.44 (2.74 to 4.14) | -1.65 (-1.97 to -1.34) | 0.31 (0.24 to 0.38) | 104.31 (83.8 to 127.36) | -1.5 (-1.82 to -1.19) |
| Myanmar | 2,589 (2,139 to 3,124) | 7.3 (6.04 to 8.72) | -0.85 (-0.92 to -0.79) | 82.05 (67.49 to 99.97) | 191.12 (158.78 to 229.9) | -1.14 (-1.21 to -1.07) |
| Philippines | 2,314 (1,801 to 2,780) | 3.72 (2.89 to 4.44) | -0.46 (-0.72 to -0.2) | 95.04 (76.13 to 114.95) | 124.48 (100.55 to 149.52) | -0.29 (-0.42 to -0.15) |
| Sri Lanka | 1,302 (943 to 1,701) | 6.41 (4.61 to 8.38) | -0.01 (-0.34 to 0.33) | 41.77 (32.4 to 51.53) | 176.98 (137.44 to 217.26) | -0.06 (-0.33 to 0.21) |
| Thailand | 3,784 (2,730 to 5,020) | 3.75 (2.71 to 4.98) | -2.41 (-2.59 to -2.23) | 152.27 (116.83 to 188.37) | 148.03 (113.72 to 183.13) | -1.6 (-1.71 to -1.49) |
| Timor Leste | 34 (25 to 43) | 5.34 (3.97 to 6.86) | 0.81 (0.71 to 0.91) | 1.17 (0.91 to 1.44) | 152.47 (119.65 to 187.49) | 0.41 (0.27 to 0.55) |
| Vietnam | 8,390 (3,392 to 11,236) | 10.94 (3.89 to 14.87) | 0.26 (0.08 to 0.45) | 235.49 (155.64 to 292.33) | 263.83 (165.55 to 328.39) | 0.26 (0.12 to 0.41) |
| Fiji | 17 (13 to 21) | 3.31 (2.58 to 4.07) | -0.23 (-0.33 to -0.12) | 0.74 (0.57 to 0.9) | 105.38 (83.32 to 126.78) | -0.15 (-0.27 to -0.03) |
| Kiribati | 2 (1 to 2) | 3.82 (2.61 to 4.8) | 1.27 (0.32 to 2.24) | 0.08 (0.06 to 0.1) | 115.58 (87.14 to 141.04) | 1 (0 to 2.01) |
| Marshall Is. | 1 (1 to 2) | 6.48 (4.79 to 8.26) | -0.19 (-0.31 to -0.08) | 0.06 (0.04 to 0.07) | 170.99 (133.3 to 209.63) | -0.21 (-0.28 to -0.15) |
| Micronesia | 4 (3 to 4) | 7.36 (5.68 to 9.06) | -0.04 (-0.11 to 0.03) | 0.12 (0.09 to 0.16) | 187.7 (146.66 to 226.81) | -0.14 (-0.24 to -0.05) |
| Papua New Guinea | 352 (146 to 510) | 12.11 (3.03 to 18.82) | 0.3 (0.17 to 0.43) | 12 (8.03 to 15.55) | 275.67 (157.37 to 368.54) | 0.36 (0.27 to 0.44) |
| Samoa | 6 (5 to 8) | 5.13 (3.84 to 6.49) | 0.02 (-0.05 to 0.08) | 0.22 (0.17 to 0.27) | 150.67 (119.77 to 181.7) | 0.08 (0.01 to 0.15) |
| Solomon Is. | 26 (21 to 33) | 12.56 (9.83 to 18.79) | 0.13 (0.01 to 0.25) | 0.97 (0.77 to 1.17) | 323.32 (263.49 to 395.1) | 0.04 (-0.09 to 0.16) |
| Tonga | 3 (2 to 3) | 3.38 (2.61 to 4.25) | -0.06 (-1.16 to 1.06) | 0.08 (0.07 to 0.1) | 106.98 (84.31 to 128.61) | 0.11 (-1.01 to 1.24) |
| Vanuatu | 7 (5 to 10) | 5.71 (4.12 to 7.67) | 0.31 (0.24 to 0.38) | 0.28 (0.21 to 0.37) | 162.3 (122.54 to 210.62) | 0.2 (0.13 to 0.27) |
| Armenia | 65 (51 to 78) | 1.66 (1.32 to 2.01) | -2.75 (-3.29 to -2.2) | 4.36 (3.31 to 5.55) | 109.45 (83.32 to 138.67) | -2.11 (-2.52 to -1.69) |
| Azerbaijan | 121 (94 to 156) | 1.52 (1.19 to 1.9) | -1.22 (-1.73 to -0.71) | 10.23 (7.74 to 12.99) | 104.1 (79.27 to 132.97) | -1 (-1.37 to -0.62) |
| Georgia | 187 (150 to 226) | 3.15 (2.52 to 3.82) | 1.97 (0.89 to 3.05) | 10.79 (8.39 to 13.51) | 191.76 (148.82 to 240.17) | 1.16 (0.55 to 1.76) |
| Kazakhstan | 419 (333 to 495) | 2.51 (2 to 2.95) | -0.02 (-0.41 to 0.37) | 29.27 (22.52 to 36.58) | 162.82 (125.04 to 203.98) | 0 (-0.25 to 0.25) |
| Kyrgyzstan | 102 (82 to 121) | 2.15 (1.74 to 2.52) | -0.65 (-0.99 to -0.3) | 6.7 (5.19 to 8.28) | 134.17 (104.27 to 167.27) | -0.82 (-1 to -0.64) |
| Mongolia | 84 (59 to 112) | 3.21 (2.35 to 4.14) | 0.01 (-0.27 to 0.3) | 5.46 (4.1 to 6.86) | 194.41 (147.72 to 243.59) | 0.42 (0.25 to 0.59) |
| Tajikistan | 85 (64 to 110) | 1.91 (1.48 to 2.4) | -0.92 (-1.25 to -0.59) | 6.44 (4.97 to 8.04) | 115.93 (90.25 to 144.27) | -1.21 (-1.51 to -0.92) |
| Turkmenistan | 37 (28 to 48) | 1.02 (0.78 to 1.28) | -2.75 (-3.02 to -2.47) | 3.77 (2.82 to 4.88) | 91.25 (68.78 to 118.13) | -1.26 (-1.45 to -1.07) |
| Uzbekistan | 452 (349 to 562) | 2.39 (1.91 to 2.86) | 0.93 (0.54 to 1.32) | 30.81 (23.3 to 38.52) | 131.17 (100.22 to 163.74) | 0.12 (-0.06 to 0.29) |
| Albania | 65 (47 to 90) | 1.59 (1.15 to 2.21) | -1.23 (-1.38 to -1.08) | 7.02 (5.09 to 9.43) | 171.29 (124.67 to 230.03) | -0.78 (-0.91 to -0.65) |
| Bosnia & Herzegovina | 132 (97 to 170) | 2.42 (1.81 to 3.08) | -1.32 (-1.5 to -1.13) | 10.6 (7.8 to 13.87) | 186.58 (137.06 to 243.74) | -0.36 (-0.45 to -0.26) |
| Bulgaria | 292 (230 to 364) | 2.11 (1.63 to 2.64) | -1.22 (-1.32 to -1.12) | 26.08 (19.42 to 34.46) | 196.28 (145.05 to 258.91) | -0.47 (-0.53 to -0.41) |
| Croatia | 712 (548 to 879) | 7.64 (5.91 to 9.44) | 0.44 (0.22 to 0.67) | 27.05 (20.8 to 33.95) | 307.34 (235.14 to 387.18) | 0.18 (0.05 to 0.31) |
| Czech Republic | 1,068 (835 to 1,291) | 4.86 (3.81 to 5.88) | -4.24 (-4.52 to -3.97) | 51.83 (38.78 to 65.91) | 253.61 (188.24 to 323.91) | -2 (-2.09 to -1.9) |
| Hungary | 1,063 (838 to 1,291) | 5.12 (4.04 to 6.23) | -4.71 (-4.99 to -4.43) | 48.08 (36.28 to 61.62) | 250.91 (188.89 to 322.99) | -2.82 (-3.02 to -2.63) |
| Macedonia | 84 (53 to 109) | 3.47 (2.12 to 4.52) | 2.07 (1.37 to 2.77) | 5.76 (4.29 to 7.48) | 192.47 (143.6 to 248.59) | 0.78 (0.55 to 1) |
| Montenegro | 29 (23 to 37) | 3.27 (2.54 to 4.12) | -0.56 (-0.69 to -0.44) | 2 (1.48 to 2.6) | 212.34 (157.83 to 275.48) | -0.22 (-0.28 to -0.17) |
| Poland | 2,912 (2,314 to 3,496) | 3.99 (3.17 to 4.79) | -2.56 (-2.73 to -2.38) | 160.27 (121.31 to 209.06) | 238.04 (179.96 to 309.78) | -1.24 (-1.31 to -1.16) |
| Romania | 870 (675 to 1,079) | 2.41 (1.86 to 2.99) | -1.98 (-2.21 to -1.75) | 71.9 (53.78 to 93.72) | 206.75 (152.91 to 267.83) | -1.3 (-1.46 to -1.14) |
| Serbia & Montenegro | 414 (321 to 512) | 2.86 (2.21 to 3.55) | -0.69 (-0.84 to -0.54) | 28.51 (21.2 to 37.07) | 191.88 (142.44 to 248.52) | -0.37 (-0.43 to -0.3) |
| Slovakia | 376 (280 to 481) | 4.25 (3.16 to 5.44) | -1.96 (-2.67 to -1.25) | 22.86 (16.81 to 29.58) | 256.61 (188.27 to 332.45) | -1.06 (-1.35 to -0.76) |
| Slovenia | 322 (235 to 412) | 6.09 (4.44 to 7.83) | -1.54 (-1.73 to -1.36) | 13.48 (10.24 to 17.36) | 302.79 (229.12 to 390.09) | -0.79 (-0.91 to -0.67) |
| Belarus | 399 (303 to 515) | 2.58 (1.94 to 3.34) | -0.79 (-1.41 to -0.16) | 33.43 (25.21 to 43.23) | 219.27 (164.83 to 284.76) | -0.41 (-0.78 to -0.03) |
| Estonia | 54 (41 to 68) | 2.01 (1.54 to 2.55) | -4.11 (-4.52 to -3.69) | 4.3 (3.24 to 5.66) | 176.98 (132.3 to 231.87) | -2.65 (-2.92 to -2.37) |
| Latvia | 118 (94 to 144) | 2.94 (2.35 to 3.61) | -3.84 (-4.3 to -3.39) | 7.53 (5.75 to 9.71) | 206.29 (156.85 to 265.62) | -2.85 (-3.13 to -2.58) |
| Lithuania | 200 (157 to 246) | 3.35 (2.62 to 4.12) | -1.41 (-1.73 to -1.09) | 12.75 (9.69 to 16.3) | 234.81 (177.65 to 299.28) | -1.29 (-1.46 to -1.13) |
| Moldova | 111 (91 to 130) | 1.96 (1.6 to 2.3) | -2.57 (-2.84 to -2.29) | 9.5 (7.26 to 12.3) | 169.31 (129.15 to 218.3) | -1.64 (-1.76 to -1.51) |
| Russia | 6,163 (4,890 to 7,360) | 2.76 (2.18 to 3.31) | -1.34 (-1.88 to -0.81) | 508.5 (381.67 to 664.45) | 227.31 (170.33 to 295.81) | -0.79 (-1.14 to -0.43) |
| Ukraine | 1,602 (1,281 to 1,918) | 2.27 (1.81 to 2.73) | -1.81 (-2.34 to -1.29) | 146.82 (111.47 to 189.71) | 206.56 (157.43 to 266.33) | -1.47 (-1.73 to -1.21) |
| Brunei | 11 (9 to 13) | 5.49 (4.58 to 6.35) | -1.17 (-1.31 to -1.04) | 0.6 (0.48 to 0.73) | 200.34 (162.26 to 241.36) | -1.12 (-1.28 to -0.96) |
| Japan | 8,921 (6,626 to 10,331) | 1.79 (1.4 to 2.02) | -1.86 (-1.97 to -1.76) | 445.61 (341.5 to 583.06) | 125.76 (95.15 to 166.47) | -0.96 (-1.05 to -0.87) |
| South Korea | 3,658 (2,897 to 4,313) | 4.45 (3.48 to 5.28) | -1.97 (-2.28 to -1.65) | 166.83 (128.84 to 211.25) | 190.55 (147.48 to 240.89) | -1.75 (-1.87 to -1.63) |
| Singapore | 91 (76 to 103) | 1.27 (1.05 to 1.44) | -1.98 (-2.27 to -1.7) | 8.65 (6.43 to 11.6) | 111.21 (82.65 to 148.4) | -0.74 (-0.85 to -0.64) |
| Australia | 2,184 (1,701 to 2,542) | 4.42 (3.49 to 5.12) | 1.17 (0.98 to 1.36) | 98.55 (75.18 to 128.57) | 231.65 (175.29 to 304.26) | 0.48 (0.4 to 0.56) |
| New Zealand | 381 (298 to 441) | 4.25 (3.35 to 4.88) | 0.02 (-0.18 to 0.22) | 19.82 (14.87 to 26.04) | 253.78 (189.77 to 335.09) | -0.04 (-0.09 to 0) |
| Andorra | 12 (9 to 16) | 7.11 (5.2 to 9.28) | -0.52 (-0.68 to -0.35) | 0.37 (0.28 to 0.47) | 247.47 (188.03 to 310.97) | -0.19 (-0.24 to -0.13) |
| Austria | 920 (747 to 1,056) | 4.2 (3.43 to 4.79) | -0.89 (-1.29 to -0.49) | 35.91 (27.94 to 45.53) | 193.47 (150.58 to 246.54) | -0.62 (-0.75 to -0.49) |
| Belgium | 1,621 (1,312 to 1,869) | 5.53 (4.55 to 6.33) | -0.03 (-0.2 to 0.14) | 59.88 (46.27 to 75.33) | 248.71 (191.83 to 314.7) | 0.56 (0.32 to 0.8) |
| Cyprus | 99 (81 to 116) | 6.2 (4.93 to 7.32) | -2.43 (-2.6 to -2.25) | 3.85 (3.05 to 4.78) | 215.06 (169.81 to 266.27) | -1.17 (-1.29 to -1.05) |
| Denmark | 568 (443 to 664) | 4.25 (3.34 to 4.96) | -3.59 (-3.88 to -3.29) | 19.15 (14.68 to 24.31) | 164.16 (125.04 to 210.94) | -2.24 (-2.43 to -2.05) |
| Finland | 744 (613 to 853) | 5.04 (4.15 to 5.73) | -1.59 (-1.75 to -1.42) | 30.53 (23.66 to 38.83) | 247.09 (190.08 to 315.26) | -0.68 (-0.95 to -0.4) |
| France | 9,997 (7,406 to 11,956) | 5.12 (3.9 to 6.04) | -1.94 (-2.04 to -1.84) | 320.7 (241.8 to 407.7) | 214.14 (161.39 to 274.23) | -0.87 (-0.93 to -0.82) |
| Germany | 9,281 (7,448 to 10,702) | 3.95 (3.18 to 4.53) | -0.86 (-1.46 to -0.25) | 345.96 (264.8 to 440.12) | 173.44 (132.12 to 221.82) | -0.46 (-0.7 to -0.23) |
| Greece | 567 (474 to 633) | 2.09 (1.78 to 2.32) | -1.92 (-2.21 to -1.64) | 30.87 (23.96 to 39.63) | 139.37 (107.5 to 178.69) | -0.99 (-1.08 to -0.9) |
| Iceland | 21 (16 to 25) | 3.1 (2.45 to 3.61) | -1.09 (-1.22 to -0.96) | 0.9 (0.68 to 1.15) | 156.68 (119.38 to 201.3) | -0.6 (-0.69 to -0.51) |
| Ireland | 200 (160 to 228) | 2.56 (2.06 to 2.92) | -2.39 (-2.69 to -2.1) | 10.76 (8.05 to 14.05) | 144.77 (108.38 to 189.43) | -0.88 (-1.03 to -0.72) |
| Israel | 348 (276 to 400) | 2.66 (2.14 to 3.04) | -1.51 (-1.73 to -1.29) | 14.93 (11.35 to 19.17) | 128.67 (98.02 to 165.31) | -0.48 (-0.58 to -0.37) |
| Italy | 7,590 (5,945 to 8,708) | 3.91 (3.11 to 4.45) | -2 (-2.1 to -1.89) | 238.11 (185.67 to 301.52) | 154.34 (119.55 to 196.94) | -1.89 (-2.13 to -1.66) |
| Luxembourg | 52 (41 to 62) | 4.41 (3.48 to 5.19) | -1.01 (-1.11 to -0.91) | 2.04 (1.57 to 2.55) | 193.41 (148.69 to 244.21) | -0.67 (-0.72 to -0.61) |
| Malta | 31 (25 to 36) | 3.07 (2.46 to 3.55) | -1.6 (-1.7 to -1.49) | 1.51 (1.15 to 1.94) | 166.7 (126.81 to 216.79) | -0.25 (-0.31 to -0.19) |
| Netherlands | 2,365 (1855 to 2,743) | 6.05 (4.77 to 7.01) | 0.9 (0.76 to 1.03) | 70.63 (55.1 to 87.9) | 197.58 (154.37 to 246.48) | 0.73 (0.4 to 1.05) |
| Norway | 736 (577 to 844) | 6.04 (4.81 to 6.87) | -0.79 (-0.89 to -0.69) | 23.01 (17.95 to 29.36) | 221.43 (171.93 to 282.84) | -0.6 (-0.64 to -0.56) |
| Portugal | 745 (618 to 845) | 2.66 (2.21 to 3) | -2.08 (-2.34 to -1.82) | 29.41 (22.85 to 37) | 123.11 (95.67 to 154.81) | -1.74 (-1.86 to -1.62) |
| Spain | 2,789 (2,252 to 3,217) | 2.32 (1.9 to 2.63) | -1.21 (-1.32 to -1.1) | 142.65 (108.1 to 183.1) | 146.88 (111.36 to 189.5) | -0.54 (-0.67 to -0.42) |
| Sweden | 1,233 (993 to 1,408) | 4.5 (3.68 to 5.09) | -0.08 (-0.28 to 0.12) | 43.71 (33.83 to 56.33) | 194.83 (150.83 to 251.73) | -0.26 (-0.39 to -0.13) |
| Switzerland | 1,268 (980 to 1,486) | 5.44 (4.31 to 6.3) | -1.26 (-1.75 to -0.76) | 43.13 (33.11 to 54.79) | 228.03 (173.96 to 292.92) | -1.16 (-1.23 to -1.08) |
| United Kingdom | 4,962 (4,047 to 5,586) | 3.33 (2.72 to 3.73) | 0.51 (0.38 to 0.64) | 199.96 (153.59 to 257.99) | 156.14 (118.93 to 201.89) | 0.35 (0.28 to 0.41) |
| Argentina | 1,572 (1,318 to 1,757) | 2.87 (2.41 to 3.21) | -1.24 (-1.37 to -1.11) | 76.35 (59.78 to 95.85) | 144.12 (112.95 to 180.8) | -0.62 (-0.67 to -0.57) |
| Chile | 958 (785 to 1,086) | 4.07 (3.32 to 4.6) | -0.05 (-0.24 to 0.14) | 39.43 (31.22 to 49.44) | 166.36 (131.73 to 208.43) | -0.41 (-0.5 to -0.32) |
| Uruguay | 246 (201 to 278) | 3.92 (3.25 to 4.39) | -0.07 (-0.24 to 0.09) | 9.19 (7.2 to 11.5) | 173.02 (134.99 to 216.44) | -0.46 (-0.55 to -0.37) |
| Canada | 3,967 (3,102 to 4,621) | 4.95 (3.94 to 5.71) | 0.16 (0.06 to 0.26) | 137.82 (106.84 to 173.6) | 193.3 (149.12 to 242.89) | 0.23 (0.17 to 0.28) |
| United States | 27,681 (23,019 to 30,871) | 4.47 (3.76 to 4.95) | 1.27 (1.09 to 1.45) | 1172.67 (926.13 to 1476.53) | 210.33 (166.33 to 263.83) | 0.22 (0.12 to 0.31) |
| Antigua & Barbuda | 3 (2 to 3) | 2.91 (2.33 to 3.45) | 0.3 (0.09 to 0.51) | 0.1 (0.08 to 0.13) | 102.33 (79.55 to 125.09) | -0.09 (-0.18 to -0.01) |
| The Bahamas | 17 (13 to 20) | 4.63 (3.62 to 5.59) | -0.87 (-1.24 to -0.5) | 0.62 (0.5 to 0.74) | 152.37 (121.91 to 182.31) | -0.76 (-1.08 to -0.45) |
| Barbados | 13 (10 to 16) | 2.75 (2.15 to 3.32) | -0.08 (-0.14 to -0.01) | 0.44 (0.34 to 0.54) | 94.19 (72.95 to 114.99) | -0.13 (-0.19 to -0.07) |
| Belize | 11 (9 to 13) | 4.1 (3.26 to 4.8) | 0 (-0.52 to 0.52) | 0.43 (0.34 to 0.51) | 146.4 (115.11 to 172.98) | 0.12 (-0.25 to 0.5) |
| Cuba | 2,008 (1,508 to 2,475) | 9.35 (7.03 to 11.5) | -0.18 (-0.38 to 0.03) | 43.92 (34.65 to 53.02) | 220.28 (173.93 to 266.35) | -0.26 (-0.42 to -0.11) |
| Dominica | 3 (2 to 4) | 3.29 (2.55 to 4.11) | -0.23 (-0.62 to 0.16) | 0.1 (0.08 to 0.12) | 112.52 (88.93 to 136.43) | -0.26 (-0.62 to 0.09) |
| Dominican Republic | 387 (286 to 507) | 4.39 (3.24 to 5.78) | 0.73 (0.55 to 0.91) | 14.61 (11.15 to 18.17) | 153.68 (117.74 to 190.84) | 0.72 (0.57 to 0.87) |
| Grenada | 4 (3 to 4) | 4.06 (3.28 to 4.67) | -0.03 (-0.2 to 0.15) | 0.14 (0.11 to 0.17) | 132.44 (105.04 to 156.2) | 0.14 (0.04 to 0.24) |
| Guyana | 35 (27 to 44) | 7.1 (5.49 to 8.73) | -0.7 (-0.87 to -0.53) | 1.22 (0.96 to 1.49) | 198.92 (157.33 to 239.95) | -0.21 (-0.28 to -0.13) |
| Haiti | 255 (179 to 381) | 4.93 (3.51 to 6.83) | -1.1 (-1.53 to -0.66) | 9.5 (6.99 to 14.25) | 137.78 (104.05 to 190.69) | -0.83 (-1.34 to -0.32) |
| Jamaica | 69 (52 to 85) | 2.03 (1.54 to 2.5) | 1.19 (0.79 to 1.58) | 2.57 (1.99 to 3.15) | 84.07 (65.1 to 103.47) | 0.69 (0.48 to 0.89) |
| Saint Lucia | 7 (5 to 8) | 3.33 (2.69 to 3.95) | -1.38 (-1.67 to -1.08) | 0.25 (0.2 to 0.3) | 115.46 (91.26 to 138.79) | -0.81 (-1 to -0.63) |
| St. Vincent & the Grenadines | 5 (4 to 6) | 4.21 (3.48 to 4.85) | 0.25 (-0.01 to 0.5) | 0.16 (0.13 to 0.19) | 123.83 (100.39 to 146.04) | 0.14 (0.01 to 0.26) |
| Suriname | 23 (18 to 28) | 4.12 (3.22 to 5.08) | -0.5 (-0.76 to -0.24) | 0.81 (0.65 to 0.98) | 135.21 (108.91 to 161.47) | -0.2 (-0.39 to -0.01) |
| Trinidad & Tobago | 42 (31 to 55) | 2.41 (1.78 to 3.13) | -2.01 (-2.21 to -1.81) | 1.71 (1.32 to 2.13) | 95.47 (73.3 to 118.25) | -0.78 (-0.95 to -0.61) |
| Bolivia | 510 (385 to 641) | 6.59 (4.98 to 8.29) | -0.45 (-0.52 to -0.38) | 15.97 (12.41 to 19.51) | 181.94 (141.44 to 221.66) | -0.68 (-0.75 to -0.62) |
| Ecuador | 800 (597 to 1,019) | 5.87 (4.45 to 7.39) | 0.22 (-0.03 to 0.47) | 26.74 (20.81 to 32.95) | 177.61 (138.94 to 218.02) | -0.03 (-0.22 to 0.15) |
| Peru | 968 (703 to 1,274) | 2.96 (2.15 to 3.9) | -0.99 (-1.21 to -0.77) | 37.62 (28.76 to 46.76) | 115.05 (88 to 142.73) | -0.59 (-0.72 to -0.46) |
| Colombia | 1,365 (1,037 to 1,741) | 2.51 (1.9 to 3.21) | -2.93 (-3.15 to -2.7) | 62.43 (48.1 to 76.67) | 117.77 (90.72 to 144.64) | -1.96 (-2.08 to -1.85) |
| Costa Rica | 281 (208 to 348) | 5.28 (3.93 to 6.56) | -1.39 (-1.6 to -1.18) | 8.41 (6.55 to 10.26) | 162.85 (126.82 to 198.62) | -0.86 (-1 to -0.71) |
| El Salvador | 361 (271 to 455) | 5.68 (4.24 to 7.19) | -1.06 (-1.3 to -0.83) | 11.26 (8.73 to 13.69) | 186.6 (144.29 to 226.98) | -1.03 (-1.21 to -0.86) |
| Guatemala | 578 (449 to 716) | 6.04 (4.78 to 7.42) | -1.29 (-1.51 to -1.06) | 21.16 (16.86 to 25.53) | 192.55 (153.98 to 232.23) | -0.6 (-0.75 to -0.46) |
| Honduras | 242 (195 to 297) | 4.42 (3.53 to 5.59) | 0.89 (0.69 to 1.09) | 9.67 (7.81 to 11.57) | 155.74 (126.78 to 185.39) | 0.23 (0.14 to 0.32) |
| Mexico | 5,002 (4,060 to 5,898) | 4.61 (3.73 to 5.43) | -2.02 (-2.3 to -1.74) | 198.85 (158.67 to 242.16) | 169.49 (136.32 to 206.39) | -0.94 (-1.21 to -0.68) |
| Nicaragua | 196 (155 to 232) | 5.73 (4.44 to 6.85) | 0.67 (0.34 to 1) | 6.32 (5.08 to 7.64) | 151.11 (121.71 to 179.65) | -0.16 (-0.28 to -0.05) |
| Panama | 101 (76 to 129) | 2.35 (1.79 to 3.01) | -2.52 (-2.78 to -2.26) | 4.57 (3.55 to 5.64) | 108.77 (84.5 to 134.11) | -1.51 (-1.66 to -1.37) |
| Venezuela | 1147 (863 to 1,466) | 4.05 (3.07 to 5.17) | -1.51 (-1.74 to -1.29) | 46.15 (35.8 to 57.03) | 155.76 (120.89 to 192.62) | -1.06 (-1.21 to -0.91) |
| Brazil | 12,135 (9,973 to 13,547) | 5.4 (4.42 to 6.05) | -0.27 (-0.51 to -0.03) | 438.93 (359.73 to 514.24) | 185.97 (152.42 to 218.07) | -0.59 (-0.71 to -0.48) |
| Paraguay | 242 (161 to 319) | 4.49 (2.95 to 5.91) | 1.43 (1.26 to 1.59) | 8.76 (6.68 to 10.77) | 155.46 (118.94 to 191.22) | 0.79 (0.69 to 0.89) |
| Algeria | 1,372 (974 to 1,744) | 4.84 (3.56 to 6.08) | -1.67 (-1.72 to -1.63) | 54.79 (40.94 to 67.51) | 158.58 (120.59 to 194.27) | -1.54 (-1.59 to -1.48) |
| Bahrain | 19 (15 to 26) | 2.76 (2.19 to 3.43) | -2.49 (-3 to -1.98) | 1.22 (0.93 to 1.53) | 100.31 (78.25 to 123.99) | -2.06 (-2.53 to -1.6) |
| Egypt | 3,405 (2,025 to 4,673) | 5.7 (3.61 to 7.69) | -0.47 (-0.59 to -0.34) | 128.27 (86.7 to 169.41) | 185.36 (127.96 to 241.32) | -0.43 (-0.52 to -0.34) |
| Iran | 3,048 (2,586 to 3,396) | 4.43 (3.77 to 4.97) | -2.21 (-2.39 to -2.03) | 117.72 (96.75 to 137.75) | 153.96 (126.93 to 180.46) | -2.08 (-2.22 to -1.94) |
| Iraq | 773 (576 to 1,025) | 3.32 (2.54 to 4.18) | -1.4 (-1.72 to -1.07) | 38.68 (29.44 to 48.98) | 145.89 (114.26 to 181.11) | -1.18 (-1.35 to -1.01) |
| Jordan | 153 (124 to 193) | 2.94 (2.38 to 3.61) | -1.99 (-2.14 to -1.83) | 7.27 (5.79 to 8.83) | 104.4 (84.44 to 126.69) | -1.58 (-1.71 to -1.46) |
| Kuwait | 76 (60 to 93) | 3.19 (2.49 to 3.88) | -1.35 (-1.79 to -0.91) | 3.75 (2.93 to 4.65) | 116.93 (92.55 to 144.11) | -1.21 (-1.5 to -0.92) |
| Lebanon | 110 (82 to 152) | 2.26 (1.63 to 3.15) | -1.39 (-1.48 to -1.29) | 4.63 (3.61 to 5.75) | 90.35 (70.59 to 112.06) | -0.82 (-0.99 to -0.64) |
| Libya | 286 (195 to 376) | 5.37 (3.69 to 6.89) | 0.27 (-0.24 to 0.77) | 11.58 (8.53 to 14.82) | 191.32 (143.31 to 239.23) | 0.21 (-0.23 to 0.65) |
| Morocco | 1,720 (1,000 to 2,449) | 6.57 (3.71 to 9.54) | -0.58 (-0.69 to -0.48) | 61.59 (44.34 to 80.7) | 197.64 (140.35 to 256.66) | -0.55 (-0.59 to -0.51) |
| Palestine | 60 (44 to 74) | 3.4 (2.39 to 4.24) | -0.8 (-1.04 to -0.56) | 2.67 (2.1 to 3.29) | 114.98 (90.24 to 140.46) | -0.53 (-0.72 to -0.33) |
| Oman | 139 (112 to 165) | 10.84 (8.81 to 12.74) | -1.39 (-1.6 to -1.18) | 5.59 (4.43 to 6.6) | 274.24 (222.3 to 319.59) | -1.9 (-2.24 to -1.57) |
| Qatar | 51 (35 to 69) | 7.5 (5.79 to 9.5) | -0.8 (-1.04 to -0.56) | 2.74 (2 to 3.49) | 206.23 (160.1 to 254.85) | -1.51 (-1.72 to -1.3) |
| Saudi Arabia | 2,335 (1,616 to 2,995) | 11.96 (9.21 to 14.55) | -0.79 (-0.94 to -0.65) | 108.87 (79.53 to 136.41) | 434.25 (329.65 to 534.32) | -0.15 (-0.3 to 0.01) |
| Syria | 307 (219 to 407) | 3.28 (2.34 to 4.22) | -0.57 (-0.7 to -0.45) | 14.31 (11.08 to 17.91) | 114.89 (89.49 to 142.2) | -0.42 (-0.55 to -0.29) |
| Tunisia | 473 (346 to 644) | 4.06 (2.99 to 5.45) | -0.79 (-0.91 to -0.67) | 18.51 (14.18 to 23.33) | 146.2 (112.51 to 183.98) | -0.65 (-0.74 to -0.57) |
| Turkey | 3,178 (1,679 to 4,250) | 3.95 (2.04 to 5.32) | 1.12 (0.61 to 1.63) | 109.11 (82.63 to 137.36) | 126.33 (95.07 to 159.11) | 0.44 (0.17 to 0.7) |
| United Arab Emirates | 367 (216 to 559) | 7.02 (4.44 to 10.07) | -1.68 (-2.21 to -1.14) | 18.39 (11.96 to 25.95) | 230.98 (156.97 to 315.3) | -1.26 (-1.58 to -0.93) |
| Yemen | 923 (611 to 1,264) | 7.15 (5.01 to 9.51) | -0.75 (-0.83 to -0.67) | 35.49 (24.72 to 47.53) | 232.77 (166.26 to 306.63) | -0.83 (-0.9 to -0.75) |
| Afghanistan | 632 (460 to 823) | 5.33 (3.95 to 6.66) | -0.67 (-0.8 to -0.54) | 26.32 (19.95 to 33.56) | 166.27 (130.75 to 203.33) | -0.61 (-0.71 to -0.5) |
| Bangladesh | 2,204 (1,456 to 2,801) | 2 (1.31 to 2.54) | -0.31 (-0.85 to 0.23) | 134.64 (101.72 to 167.67) | 103.27 (78.48 to 128.74) | -0.22 (-0.46 to 0.03) |
| Bhutan | 50 (30 to 69) | 11.37 (6.6 to 15.82) | 1.29 (1.03 to 1.55) | 1.46 (1.08 to 1.82) | 281.01 (204.14 to 354.94) | 0.65 (0.52 to 0.78) |
| India | 118,682 (94,278 to 140,775) | 13.71 (10.83 to 16.24) | -1.11 (-1.32 to -0.91) | 3,988.26 (3,259.83 to 4,777) | 381.76 (311.84 to 454.05) | -0.46 (-0.56 to -0.36) |
| Nepal | 1,599 (1,093 to 3,014) | 9.49 (6.27 to 19.44) | 0.45 (0.34 to 0.57) | 54.66 (42.59 to 74.75) | 265.57 (204.28 to 383.19) | 0.09 (-0.05 to 0.22) |
| Pakistan | 3,302 (2,345 to 4,364) | 3.97 (2.67 to 5.35) | -0.08 (-0.27 to 0.11) | 160.26 (127.22 to 198.82) | 143.37 (113.25 to 177.2) | 0.24 (0.15 to 0.32) |
| Angola | 718 (560 to 959) | 8.27 (6.64 to 10.72) | -0.44 (-0.58 to -0.3) | 26.21 (20.38 to 33.92) | 222.99 (179.22 to 281.84) | -0.57 (-0.7 to -0.44) |
| Central African Republic | 213 (153 to 289) | 10.6 (7.9 to 14.09) | -0.12 (-0.23 to 0) | 7.82 (5.82 to 10.4) | 304.35 (233.61 to 399.33) | 0.04 (-0.09 to 0.18) |
| Congo | 175 (134 to 223) | 8.24 (6.58 to 10.06) | -1.1 (-1.28 to -0.92) | 6.15 (4.67 to 7.81) | 216.73 (174.4 to 264.29) | -1.18 (-1.37 to -0.99) |
| Congo, DRC | 2,597 (1,931 to 3,624) | 8.76 (6.73 to 11.84) | -0.34 (-0.38 to -0.29) | 89.43 (67.81 to 119.82) | 237.93 (184.2 to 312.62) | -0.25 (-0.3 to -0.21) |
| Equatorial Guinea | 25 (18 to 37) | 6.89 (5.04 to 9.67) | -1.19 (-1.43 to -0.95) | 0.85 (0.63 to 1.19) | 177.52 (136.32 to 244.45) | -1.46 (-1.72 to -1.21) |
| Gabon | 72 (56 to 92) | 8.19 (6.4 to 10.52) | -0.74 (-0.87 to -0.62) | 2.35 (1.82 to 2.95) | 220 (175.25 to 271.36) | -0.71 (-0.86 to -0.56) |
| Burundi | 265 (205 to 345) | 7.92 (6.27 to 9.99) | -0.96 (-1.08 to -0.84) | 9.53 (7.58 to 12.05) | 216.13 (174.77 to 265.36) | -0.86 (-0.95 to -0.77) |
| Comoros | 33 (26 to 40) | 7.8 (6.31 to 9.58) | -0.31 (-0.92 to 0.31) | 1.12 (0.92 to 1.35) | 232.31 (191.37 to 278.69) | -0.32 (-0.96 to 0.31) |
| Djibouti | 39 (31 to 48) | 8.31 (6.81 to 10.3) | 0.18 (-0.13 to 0.48) | 1.51 (1.25 to 1.84) | 238.89 (199.45 to 288.01) | 0.04 (-0.27 to 0.36) |
| Eritrea | 170 (129 to 221) | 8.85 (6.89 to 11.3) | 0.2 (0.03 to 0.37) | 6.56 (5.19 to 8.29) | 245.56 (197.94 to 304.82) | -0.04 (-0.15 to 0.07) |
| Ethiopia | 2,368 (2,002 to 2,733) | 7.64 (6.38 to 8.84) | -1.28 (-1.36 to -1.2) | 74.02 (61.75 to 86.26) | 191.41 (161.39 to 221.61) | -1.59 (-1.72 to -1.47) |
| Kenya | 1,144 (964 to 1314) | 7.43 (6.14 to 8.48) | 0.36 (0.23 to 0.49) | 38.52 (32.44 to 45.11) | 188.95 (160.57 to 218.71) | 0.17 (0.05 to 0.28) |
| Madagascar | 439 (335 to 558) | 5.68 (4.43 to 7) | -0.59 (-0.7 to -0.47) | 18.8 (15.07 to 22.96) | 174.44 (141.59 to 211.01) | -0.44 (-0.51 to -0.37) |
| Malawi | 391 (321 to 462) | 7.23 (5.91 to 8.5) | -0.38 (-0.48 to -0.27) | 12.86 (10.62 to 15.17) | 189.48 (157.84 to 220.38) | -0.32 (-0.41 to -0.22) |
| Mauritius | 39 (31 to 47) | 2.36 (1.88 to 2.88) | -0.13 (-0.3 to 0.03) | 1.82 (1.44 to 2.2) | 105.39 (83.41 to 127.21) | -0.03 (-0.13 to 0.08) |
| Mozambique | 828 (635 to 1,034) | 10.03 (7.82 to 12.38) | 0.54 (0.46 to 0.63) | 29.2 (23.39 to 35.26) | 277.29 (224.75 to 331.35) | 0.56 (0.49 to 0.63) |
| Rwanda | 385 (321 to 469) | 8.74 (7.3 to 10.41) | -1.57 (-1.8 to -1.34) | 13.31 (10.94 to 15.92) | 235.5 (195.81 to 278.12) | -1.72 (-1.95 to -1.48) |
| Seychelles | 3 (3 to 4) | 3.36 (2.78 to 3.88) | -0.55 (-0.64 to -0.46) | 0.15 (0.12 to 0.17) | 127.81 (104.15 to 149.89) | -0.44 (-0.52 to -0.37) |
| Somalia | 423 (308 to 565) | 8.4 (6.3 to 10.98) | 0 (-0.09 to 0.08) | 16.24 (12.46 to 21.08) | 237.86 (185.42 to 299.37) | -0.05 (-0.12 to 0.02) |
| Tanzania | 1,261 (1,063 to 1,478) | 6.71 (5.66 to 7.85) | -0.19 (-0.25 to -0.13) | 42.9 (35.6 to 50.32) | 183.88 (154.84 to 212.73) | -0.21 (-0.29 to -0.13) |
| Uganda | 864 (697 to 1,031) | 8.21 (6.65 to 9.7) | -0.27 (-0.44 to -0.11) | 28.36 (22.86 to 33.54) | 213.16 (173.85 to 248.48) | -0.25 (-0.4 to -0.1) |
| Zambia | 398 (314 to 490) | 8.17 (6.63 to 9.9) | -0.58 (-0.78 to -0.39) | 13.56 (10.77 to 16.47) | 211.94 (173.69 to 251.82) | -0.48 (-0.64 to -0.32) |
| Botswana | 60 (43 to 80) | 4.72 (3.47 to 6.14) | -0.51 (-0.92 to -0.1) | 2.48 (1.82 to 3.24) | 162.35 (122.99 to 205.97) | -0.26 (-0.6 to 0.08) |
| Lesotho | 95 (70 to 122) | 8.02 (6.03 to 10.27) | 1.36 (1.06 to 1.66) | 3.56 (2.68 to 4.51) | 259.66 (198.22 to 325.08) | 1.43 (1.16 to 1.7) |
| Namibia | 73 (56 to 94) | 5.52 (4.3 to 6.96) | -0.68 (-0.99 to -0.38) | 2.71 (2.07 to 3.47) | 180.1 (140.23 to 226.03) | -0.63 (-0.92 to -0.34) |
| South Africa | 1,821 (1,553 to 2,046) | 4.24 (3.61 to 4.77) | -1.52 (-1.96 to -1.08) | 73.71 (60.97 to 84.55) | 155.48 (129.84 to 178.35) | -1.45 (-1.74 to -1.15) |
| Eswatini | 34 (23 to 47) | 6.22 (4.38 to 8.29) | 0.13 (-0.34 to 0.6) | 1.34 (0.98 to 1.8) | 207.66 (154.15 to 269.93) | 0.29 (-0.16 to 0.74) |
| Zimbabwe | 438 (340 to 549) | 8.15 (6.24 to 10.23) | -0.16 (-0.29 to -0.04) | 14.49 (11.43 to 18.13) | 206.59 (166.12 to 249.5) | -0.25 (-0.34 to -0.17) |
| Benin | 290 (226 to 369) | 7.58 (6 to 9.45) | -0.64 (-0.75 to -0.53) | 9.8 (7.86 to 12.25) | 206.69 (169.06 to 253.56) | -0.48 (-0.57 to -0.4) |
| Burkina Faso | 706 (569 to 851) | 9.83 (8.12 to 11.63) | -0.34 (-0.47 to -0.22) | 23.14 (18.49 to 27.68) | 259.2 (211.52 to 305.47) | -0.09 (-0.25 to 0.08) |
| Cameroon | 843 (653 to 1,107) | 9.11 (7.26 to 11.5) | -0.44 (-0.61 to -0.28) | 29.26 (23 to 37.11) | 249.4 (200.61 to 310.73) | -0.27 (-0.41 to -0.12) |
| Cape Verde | 23 (18 to 29) | 5.36 (4.34 to 6.81) | 0.63 (0.24 to 1.02) | 0.65 (0.54 to 0.77) | 151.16 (126.13 to 178.55) | 0.55 (0.24 to 0.86) |
| Chad | 348 (278 to 431) | 7.89 (6.41 to 9.59) | 0.04 (-0.14 to 0.22) | 11.63 (9.48 to 14.05) | 213.21 (175.56 to 254.33) | 0.22 (0.09 to 0.36) |
| Cote dIvoire | 584 (467 to 723) | 7.69 (6.26 to 9.35) | -0.63 (-0.85 to -0.41) | 20.88 (16.82 to 25.33) | 206.26 (169.27 to 244.31) | -0.53 (-0.75 to -0.31) |
| The Gambia | 64 (52 to 78) | 8.49 (6.84 to 10.16) | 0.05 (-0.14 to 0.24) | 1.88 (1.52 to 2.23) | 208.97 (169.37 to 246.41) | 0 (-0.22 to 0.22) |
| Ghana | 874 (722 to 1,066) | 7.06 (5.89 to 8.49) | -0.23 (-0.36 to -0.11) | 32.78 (26.76 to 39) | 207.76 (172.32 to 245.24) | 0 (-0.08 to 0.08) |
| Guinea | 363 (288 to 443) | 7.84 (6.26 to 9.54) | 0.01 (-0.13 to 0.15) | 11.56 (9.41 to 13.75) | 216.24 (178.12 to 255.97) | 0.19 (0.07 to 0.31) |
| Guinea-Bissau | 47 (37 to 59) | 8.41 (6.6 to 10.57) | -0.5 (-0.74 to -0.25) | 1.8 (1.44 to 2.18) | 243.52 (198.08 to 290.87) | -0.46 (-0.71 to -0.21) |
| Liberia | 103 (81 to 132) | 6.66 (5.28 to 8.44) | -0.73 (-0.84 to -0.62) | 3.39 (2.71 to 4.21) | 169.78 (138.97 to 208.01) | -0.57 (-0.66 to -0.49) |
| Mali | 513 (380 to 686) | 8.03 (5.91 to 10.79) | -0.46 (-0.5 to -0.43) | 16.13 (12.91 to 20.52) | 203.45 (162.76 to 258.73) | -0.39 (-0.44 to -0.34) |
| Mauritania | 123 (94 to 161) | 6.98 (5.36 to 9.02) | -1.38 (-1.48 to -1.29) | 4.22 (3.31 to 5.16) | 205.17 (163.41 to 248.23) | -1.27 (-1.42 to -1.12) |
| Niger | 457 (337 to 623) | 8.58 (6.24 to 11.87) | -0.24 (-0.27 to -0.21) | 15.29 (12.06 to 19.6) | 216.33 (171.73 to 276.42) | -0.26 (-0.31 to -0.21) |
| Nigeria | 3,922 (2,975 to 5,049) | 6.3 (4.82 to 8.14) | 0.1 (0 to 0.19) | 129.22 (104.19 to 158.2) | 161.27 (130.2 to 195.61) | -0.13 (-0.17 to -0.08) |
| Sao Tome & Principe | 7 (6 to 9) | 9.39 (7.29 to 12.06) | 0.73 (0.53 to 0.93) | 0.23 (0.19 to 0.27) | 232.13 (190.77 to 276.29) | 0.6 (0.37 to 0.84) |
| Senegal | 432 (344 to 530) | 7.39 (6.01 to 8.93) | -0.17 (-0.41 to 0.06) | 13.58 (10.96 to 16.35) | 193.26 (158.48 to 231.59) | -0.1 (-0.36 to 0.16) |
| Sierra Leone | 215 (170 to 272) | 7.53 (6.04 to 9.28) | -0.32 (-0.51 to -0.14) | 7.15 (5.77 to 8.89) | 204.52 (167.06 to 250.87) | -0.13 (-0.31 to 0.04) |
| Togo | 186 (148 to 235) | 6.76 (5.46 to 8.44) | -0.37 (-0.45 to -0.29) | 7.34 (5.92 to 8.99) | 198.4 (163.18 to 239.26) | -0.19 (-0.23 to -0.14) |
| American Samoa | 2 (1 to 2) | 4.34 (3.45 to 5.28) | -0.18 (-0.33 to -0.04) | 0.06 (0.05 to 0.07) | 129.89 (104.18 to 154.14) | -0.33 (-0.45 to -0.21) |
| Bermuda | 3 (3 to 4) | 2.52 (2.01 to 3.06) | -1.78 (-2 to -1.56) | 0.13 (0.1 to 0.16) | 103.51 (80.23 to 126.93) | -1.09 (-1.31 to -0.87) |
| Cook Is. | 1 (1 to 1) | 3.5 (2.79 to 4.26) | -1.4 (-1.52 to -1.28) | 0.03 (0.03 to 0.04) | 136.87 (108.38 to 165.06) | -0.92 (-1.07 to -0.76) |
| Greenland | 5 (4 to 6) | 10.5 (8.23 to 12.73) | -1.52 (-1.72 to -1.33) | 0.21 (0.17 to 0.26) | 347.46 (275.36 to 421.83) | -1.37 (-1.48 to -1.26) |
| Guam | 5 (4 to 5) | 2.42 (1.91 to 2.92) | -3.16 (-5.75 to -0.49) | 0.2 (0.15 to 0.24) | 104 (81.79 to 126.59) | -1.72 (-3.8 to 0.4) |
| Monaco | 3 (2 to 3) | 2.27 (1.71 to 2.76) | -0.44 (-0.48 to -0.4) | 0.14 (0.1 to 0.18) | 146.51 (109.75 to 191.63) | 0.11 (0.06 to 0.17) |
| Nauru | 0 (0 to 0) | 6.46 (4.93 to 7.89) | 0.09 (-0.31 to 0.49) | 0.01 (0.01 to 0.01) | 192.79 (151.15 to 232.71) | 0.05 (-0.22 to 0.32) |
| Niue | 0 (0 to 0) | 4.37 (3.19 to 5.56) | -0.46 (-0.54 to -0.39) | 0 (0 to 0) | 137.5 (108.32 to 167.02) | -0.27 (-0.31 to -0.23) |
| Northern Mariana Is. | 2 (1 to 2) | 5.33 (4.3 to 6.34) | -0.51 (-0.58 to -0.44) | 0.08 (0.06 to 0.09) | 161.38 (129.35 to 192.72) | -0.75 (-0.83 to -0.67) |
| Palau | 1 (1 to 1) | 8.17 (6.32 to 10.17) | -0.57 (-0.69 to -0.45) | 0.05 (0.04 to 0.06) | 231.93 (185.79 to 282.46) | -0.28 (-0.37 to -0.19) |
| Puerto Rico | 219 (162 to 275) | 2.69 (2.01 to 3.41) | -1.32 (-1.56 to -1.09) | 8.19 (6.33 to 10.14) | 119.24 (92.02 to 148.83) | -0.6 (-0.69 to -0.52) |
| St. Kitts & Nevis | 2 (2 to 3) | 4.21 (3.36 to 5) | -0.79 (-0.95 to -0.64) | 0.08 (0.07 to 0.1) | 133.85 (103.99 to 162.06) | -0.98 (-1.11 to -0.84) |
| San Marino | 2 (2 to 3) | 2.91 (1.95 to 3.98) | -0.36 (-0.48 to -0.23) | 0.1 (0.08 to 0.13) | 159.31 (119.64 to 203.32) | 0.03 (-0.05 to 0.11) |
| Tokelau | 0 (0 to 0) | 4.37 (3.23 to 5.61) | -0.11 (-0.22 to -0.01) | 0 (0 to 0) | 132.75 (103.79 to 160.95) | -0.13 (-0.16 to -0.1) |
| Tuvalu | 0 (0 to 1) | 5.61 (4.15 to 7.29) | -0.32 (-0.35 to -0.28) | 0.02 (0.01 to 0.02) | 162.56 (127.03 to 201.48) | -0.27 (-0.34 to -0.19) |
| Virgin Is. | 7 (5 to 8) | 4.25 (3.34 to 5.09) | 0.1 (-0.07 to 0.27) | 0.23 (0.18 to 0.27) | 136.25 (108.83 to 162.9) | 0.01 (-0.09 to 0.12) |
| South Sudan | 175 (133 to 229) | 6.02 (4.65 to 7.75) | -0.45 (-0.49 to -0.41) | 6.88 (5.45 to 8.67) | 180.28 (144.23 to 223.32) | -0.46 (-0.51 to -0.41) |
| Sudan | 922 (603 to 1,251) | 5.51 (3.67 to 7.36) | -0.95 (-0.98 to -0.91) | 33.61 (24.01 to 43.53) | 171.03 (123.56 to 217.28) | -0.9 (-0.94 to -0.86) |

Data are presented with 95% uncertainty interval. For EAPC, data are presented with EAPC value with 95% confidence interval. SDI, socio-demographic index; EAPC, estimated annual percentage change.
