# Supplementary material for: Global, Regional, and National Burden of Low Bone Mineral Density From 1990 to 2019: Results From the Global Burden of Disease Study 2019
Source: Front Endocrinol (Lausanne). 2022 May 24;13:870905. doi: 10.3389/fendo.2022.870905 (PMC9172621; doi:10.3389/fendo.2022.870905)
Supplement: Supplementary file 9 [file Table_3.docx]

**Supplementary material 3.** an example to calculate the SDI for a country X with Mean educ yrs pc of 8.23, TFU25 of 1.09 and ln (LDI) of 9.6


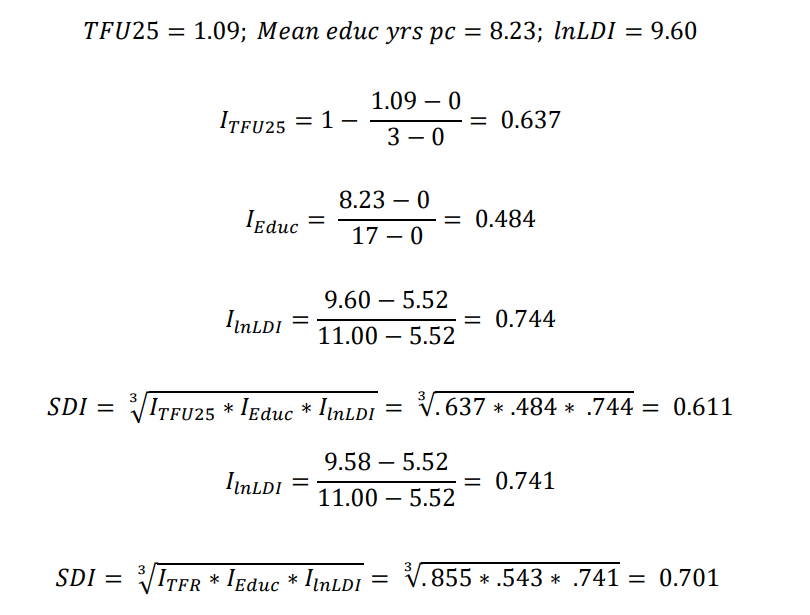


The socio-demographic index of each country or territory and their category in 2019 are provided in the table below

| **Location** | **Socio-demographic index** | **Category** |
| --- | --- | --- |
| China | 0.686 | Middle SDI |
| North Korea | 0.558 | Low-middle SDI |
| Taiwan (Province of China) | 0.868 | High SDI |
| Cambodia | 0.469 | Low-middle SDI |
| Indonesia | 0.66 | Middle SDI |
| Laos | 0.49 | Low-middle SDI |
| Malaysia | 0.737 | High-middle SDI |
| Maldives | 0.562 | Low-middle SDI |
| Myanmar | 0.521 | Low-middle SDI |
| Philippines | 0.623 | Middle SDI |
| Sri Lanka | 0.69 | High-middle SDI |
| Thailand | 0.687 | Middle SDI |
| Timor Leste | 0.514 | Low-middle SDI |
| Vietnam | 0.617 | Middle SDI |
| Fiji | 0.664 | Middle SDI |
| Kiribati | 0.527 | Low-middle SDI |
| Marshall Is. | 0.544 | Low-middle SDI |
| Micronesia | 0.58 | Low-middle SDI |
| Papua New Guinea | 0.394 | Low SDI |
| Samoa | 0.641 | Middle SDI |
| Solomon Is. | 0.407 | Low SDI |
| Tonga | 0.636 | Middle SDI |
| Vanuatu | 0.485 | Low-middle SDI |
| Armenia | 0.689 | Middle SDI |
| Azerbaijan | 0.683 | Middle SDI |
| Georgia | 0.702 | High-middle SDI |
| Kazakhstan | 0.723 | High-middle SDI |
| Kyrgyzstan | 0.596 | Low-middle SDI |
| Mongolia | 0.606 | Low-middle SDI |
| Tajikistan | 0.539 | Low-middle SDI |
| Turkmenistan | 0.67 | Middle SDI |
| Uzbekistan | 0.631 | Middle SDI |
| Albania | 0.681 | Middle SDI |
| Bosnia & Herzegovina | 0.718 | High-middle SDI |
| Bulgaria | 0.764 | High-middle SDI |
| Croatia | 0.794 | High-middle SDI |
| Czech Republic | 0.828 | High SDI |
| Hungary | 0.791 | High-middle SDI |
| Macedonia | 0.744 | High-middle SDI |
| Montenegro | 0.791 | High-middle SDI |
| Poland | 0.802 | High-middle SDI |
| Romania | 0.76 | High-middle SDI |
| Serbia & Montenegro | 0.767 | High-middle SDI |
| Slovakia | 0.812 | High SDI |
| Slovenia | 0.84 | High SDI |
| Belarus | 0.745 | High-middle SDI |
| Estonia | 0.835 | High SDI |
| Latvia | 0.82 | High SDI |
| Lithuania | 0.843 | High SDI |
| Moldova | 0.696 | High-middle SDI |
| Russia | 0.805 | High-middle SDI |
| Ukraine | 0.736 | High-middle SDI |
| Brunei | 0.823 | High SDI |
| Japan | 0.87 | High SDI |
| South Korea | 0.878 | High SDI |
| Singapore | 0.861 | High SDI |
| Australia | 0.839 | High SDI |
| New Zealand | 0.84 | High SDI |
| Andorra | 0.894 | High SDI |
| Austria | 0.849 | High SDI |
| Belgium | 0.851 | High SDI |
| Cyprus | 0.841 | High SDI |
| Denmark | 0.89 | High SDI |
| Finland | 0.856 | High SDI |
| France | 0.834 | High SDI |
| Germany | 0.898 | High SDI |
| Greece | 0.794 | High-middle SDI |
| Iceland | 0.869 | High SDI |
| Ireland | 0.867 | High SDI |
| Israel | 0.803 | High-middle SDI |
| Italy | 0.801 | High-middle SDI |
| Luxembourg | 0.895 | High SDI |
| Malta | 0.801 | High-middle SDI |
| Netherlands | 0.883 | High SDI |
| Norway | 0.913 | High SDI |
| Portugal | 0.743 | High-middle SDI |
| Spain | 0.767 | High-middle SDI |
| Sweden | 0.872 | High SDI |
| Switzerland | 0.929 | High SDI |
| United Kingdom | 0.847 | High SDI |
| Argentina | 0.708 | High-middle SDI |
| Chile | 0.759 | High-middle SDI |
| Uruguay | 0.697 | High-middle SDI |
| Canada | 0.873 | High SDI |
| United States | 0.859 | High SDI |
| Antigua & Barbuda | 0.743 | High-middle SDI |
| The Bahamas | 0.796 | High-middle SDI |
| Barbados | 0.742 | High-middle SDI |
| Belize | 0.603 | Low-middle SDI |
| Cuba | 0.668 | Middle SDI |
| Dominica | 0.729 | High-middle SDI |
| Dominican Republic | 0.592 | Low-middle SDI |
| Grenada | 0.669 | Middle SDI |
| Guyana | 0.618 | Middle SDI |
| Haiti | 0.432 | Low SDI |
| Jamaica | 0.684 | Middle SDI |
| Saint Lucia | 0.67 | Middle SDI |
| St. Vincent & the Grenadines | 0.627 | Middle SDI |
| Suriname | 0.636 | Middle SDI |
| Trinidad & Tobago | 0.757 | High-middle SDI |
| Bolivia | 0.566 | Low-middle SDI |
| Ecuador | 0.64 | Middle SDI |
| Peru | 0.648 | Middle SDI |
| Colombia | 0.633 | Middle SDI |
| Costa Rica | 0.68 | Middle SDI |
| El Salvador | 0.573 | Low-middle SDI |
| Guatemala | 0.526 | Low-middle SDI |
| Honduras | 0.496 | Low-middle SDI |
| Mexico | 0.649 | Middle SDI |
| Nicaragua | 0.517 | Low-middle SDI |
| Panama | 0.686 | Middle SDI |
| Venezuela | 0.686 | Middle SDI |
| Brazil | 0.64 | Middle SDI |
| Paraguay | 0.638 | Middle SDI |
| Algeria | 0.652 | Middle SDI |
| Bahrain | 0.751 | High-middle SDI |
| Egypt | 0.658 | Middle SDI |
| Iran | 0.67 | Middle SDI |
| Iraq | 0.671 | Middle SDI |
| Jordan | 0.731 | High-middle SDI |
| Kuwait | 0.851 | High SDI |
| Lebanon | 0.708 | High-middle SDI |
| Libya | 0.709 | High-middle SDI |
| Morocco | 0.548 | Low-middle SDI |
| Palestine | 0.588 | Low-middle SDI |
| Oman | 0.783 | High-middle SDI |
| Qatar | 0.83 | High SDI |
| Saudi Arabia | 0.805 | High-middle SDI |
| Syria | 0.619 | Middle SDI |
| Tunisia | 0.672 | Middle SDI |
| Turkey | 0.748 | High-middle SDI |
| United Arab Emirates | 0.88 | High SDI |
| Yemen | 0.412 | Low SDI |
| Afghanistan | 0.343 | Low SDI |
| Bangladesh | 0.483 | Low-middle SDI |
| Bhutan | 0.455 | Low-middle SDI |
| India | 0.566 | Low-middle SDI |
| Nepal | 0.422 | Low SDI |
| Pakistan | 0.449 | Low SDI |
| Angola | 0.47 | Low-middle SDI |
| Central African Republic | 0.274 | Low SDI |
| Congo | 0.568 | Low-middle SDI |
| Congo, DRC | 0.382 | Low SDI |
| Equatorial Guinea | 0.685 | Middle SDI |
| Gabon | 0.656 | Middle SDI |
| Burundi | 0.284 | Low SDI |
| Comoros | 0.455 | Low-middle SDI |
| Djibouti | 0.459 | Low-middle SDI |
| Eritrea | 0.396 | Low SDI |
| Ethiopia | 0.343 | Low SDI |
| Kenya | 0.508 | Low-middle SDI |
| Madagascar | 0.396 | Low SDI |
| Malawi | 0.384 | Low SDI |
| Mauritius | 0.705 | High-middle SDI |
| Mozambique | 0.307 | Low SDI |
| Rwanda | 0.429 | Low SDI |
| Seychelles | 0.724 | High-middle SDI |
| Somalia | 0.081 | Low SDI |
| Tanzania | 0.423 | Low SDI |
| Uganda | 0.404 | Low SDI |
| Zambia | 0.505 | Low-middle SDI |
| Botswana | 0.634 | Middle SDI |
| Lesotho | 0.507 | Low-middle SDI |
| Namibia | 0.612 | Middle SDI |
| South Africa | 0.678 | Middle SDI |
| Eswatini | 0.577 | Low-middle SDI |
| Zimbabwe | 0.476 | Low-middle SDI |
| Benin | 0.352 | Low SDI |
| Burkina Faso | 0.257 | Low SDI |
| Cameroon | 0.49 | Low-middle SDI |
| Cape Verde | 0.525 | Low-middle SDI |
| Chad | 0.238 | Low SDI |
| Cote dIvoire | 0.408 | Low SDI |
| The Gambia | 0.399 | Low SDI |
| Ghana | 0.557 | Low-middle SDI |
| Guinea | 0.325 | Low SDI |
| Guinea-Bissau | 0.355 | Low SDI |
| Liberia | 0.37 | Low SDI |
| Mali | 0.263 | Low SDI |
| Mauritania | 0.496 | Low-middle SDI |
| Niger | 0.162 | Low SDI |
| Nigeria | 0.515 | Low-middle SDI |
| Sao Tome & Principe | 0.5 | Low-middle SDI |
| Senegal | 0.389 | Low SDI |
| Sierra Leone | 0.347 | Low SDI |
| Togo | 0.417 | Low SDI |
| American Samoa | 0.712 | High-middle SDI |
| Bermuda | 0.813 | High SDI |
| Cook Is. | 0.764 | High-middle SDI |
| Greenland | 0.761 | High-middle SDI |
| Guam | 0.813 | High SDI |
| Monaco | 0.902 | High SDI |
| Nauru | 0.618 | Middle SDI |
| Niue | 0.711 | High-middle SDI |
| Northern Mariana Is. | 0.771 | High-middle SDI |
| Palau | 0.738 | High-middle SDI |
| Puerto Rico | 0.814 | High SDI |
| St. Kitts & Nevis | 0.746 | High-middle SDI |
| San Marino | 0.884 | High SDI |
| Tokelau | 0.626 | Middle SDI |
| Tuvalu | 0.589 | Low-middle SDI |
| Virgin Is. | 0.799 | High-middle SDI |
| South Sudan | 0.363 | Low SDI |
| Sudan | 0.515 | Low-middle SDI |
